# Global diversity and biogeography of DNA viral communities in activated sludge systems

**DOI:** 10.1186/s40168-023-01672-1

**Published:** 2023-10-21

**Authors:** Xiangyu Fan, Mengzhi Ji, Dashuai Mu, Xianghe Zeng, Zhen Tian, Kaili Sun, Rongfeng Gao, Yang Liu, Xinyuan He, Linwei Wu, Qiang Li

**Affiliations:** 1 School of Biological Science and Technology, University of Jinan, Jinan, Shandong Province China; 2 Artificial Intelligence Institute, University of Jinan, Jinan, Shandong Province China; 3 Institute of Marine Science and Technology, Shandong University, Qingdao, Shandong Province China; 4 State Key Laboratory of Microbial Technology, Institute of Microbial Technology, Shandong University, Qingdao, Shandong Province China; 5 Marine College, Shandong University, Weihai, Shandong Province China; 6 Institute of Ecology, Key Laboratory for Earth Surface Processes of the Ministry of Education, College of Urban and Environmental Sciences, Peking University, Beijing, China

**Keywords:** Activated sludge, Viral community, Biogeography, Auxiliary metabolic genes, Virus-microbe interaction

## Abstract

**Background:**

Activated sludge (AS) systems in wastewater treatment plants (WWTPs) harbor enormous viruses that regulate microbial metabolism and nutrient cycling, significantly influencing the stability of AS systems. However, our knowledge about the diversity of viral taxonomic groups and functional traits in global AS systems is still limited. To address this gap, we investigated the global diversity and biogeography of DNA viral communities in AS systems using 85,114 viral operational taxonomic units (vOTUs) recovered from 144 AS samples collected across 54 WWTPs from 13 different countries.

**Results:**

AS viral communities and their functional traits exhibited distance-decay relationship (DDR) at the global scale and latitudinal diversity gradient (LDG) from equator to mid-latitude. Furthermore, it was observed that AS viral community and functional gene structures were largely driven by the geographic factors and wastewater types, of which the geographic factors were more important. Carrying and disseminating auxiliary metabolic genes (AMGs) associated with the degradation of polysaccharides, sulfate reduction, denitrification, and organic phosphoester hydrolysis, as well as the lysis of crucial functional microbes that govern biogeochemical cycles were two major ways by which viruses could regulate AS functions. It was worth noting that our study revealed a high abundance of antibiotic resistance genes (ARGs) in viral genomes, suggesting that viruses were key reservoirs of ARGs in AS systems.

**Conclusions:**

Our results demonstrated the highly diverse taxonomic groups and functional traits of viruses in AS systems. Viral lysis of host microbes and virus-mediated HGT can regulate the biogeochemical and nutrient cycles, thus affecting the performance of AS systems. These findings provide important insights into the viral diversity, function, and ecology in AS systems on a global scale.

Video Abstract

**Supplementary Information:**

The online version contains supplementary material available at 10.1186/s40168-023-01672-1.

## Background

Activated sludge (AS) systems are utilized at more than 80% of wastewater treatment plants (WWTPs), making it the most widely used wastewater biological treatment technology [1]. As one of the most complex artificial microbial communities, AS is formed by various microbes, including prokaryotes, fungi, protozoa, and viruses, together with their adsorbed organic and inorganic substances [1]. The complex and incompletely defined microbial communities in AS are able to remove carbon (C), nitrogen (N), phosphorus (P), and pathogens, and drive biotransformation of micropollutants in wastewater [2–4]. Hence, an in-depth study of the diversity and functional mechanisms of microbial communities in AS is beneficial for improving wastewater treatment technology. In this context, a great deal of effort has been devoted to AS microbial ecology, including advances in the study of the global diversity and ecological mechanism of bacterial communities in WWTPs [2, 4–6]. However, the roles of viruses and the diversity of the viral community are just beginning to be understood in WWTPs.

Viruses, a group of non-cellular organisms, are the most abundant living entity on Earth, but depend on host cells for their own propagation [7]. The crucial roles of viruses in some ecosystems, including marine and human-associated samples, have been revealed [8]. By infecting and lysing host cells, viruses can affect the composition of microbial populations and facilitate the transfer of organic matter from cells to the dissolved organic matter pool via the “viral shunt” [9], and when consumed by small phagotrophs, viruses facilitate the movement of organic matter into the classical food web, which is called the “viral sweep” [10]. In addition, viruses have been shown to regulate the metabolic processes of ecosystems by encoding auxiliary metabolic genes (AMGs) [11]. Viruses are also considered to be potential reservoirs of antibiotic resistance genes (ARGs) and the delivery agents for horizontal gene transfer (HGT) of ARGs in the environment [7, 12].

AS systems of WWTPs are hotspots for viruses, with viral concentrations higher than those of oceans, lakes, soil, and other natural ecosystems [13]. While some research groups have studied the diversity and function of the viral community in WWTPs, the majority have only focused on specific geographic regions or clusters of several plants. For instance, Zhang et al. found viruses could control prokaryotic communities in the anaerobic digesters of three WWTPs located in China (40.6% of total variations) [14]. Shi et al. studied three conventional WWTPs located in Taiwan and found that viruses encoded AMGs associated with the removal of nutrients and pollutants, as well as ARGs which were detrimental to human health [15]. Chen et al.’s study of six biological WWTPs in Hong Kong revealed that viruses can regulate microbial taxonomic communities and functional structures through lytic and lysogenic processes, thus affecting nutrient removal and biogeochemical cycling in AS [16]. Li et al. analyzed the metagenomic and viromic data from 27 WWTPs and evaluated viral characteristics and viral removal efficacy by AS treatment [17]. Using samples at a WWTP in Singapore, Gu et al. developed a fluorescence-activated cell sorting (FACS)-coupled metagenomic sequencing strategy which could improve the detection of viruses from sequencing data [18]. However, so far, the global diversity and distribution of viral communities in WWTPs still lack a systematic description.

Viral communities and their functions have shown geographic distribution patterns in some environments, such as the ocean [19, 20], human gut [21, 22], and drinking water [23]. Based on global ocean viral communities, five ecological zones were revealed [20]. Temperature was found to be the most critical factor in structuring these ecological zones. Except in the Arctic region, both ocean viral macro- and micro diversity were observed to follow the latitudinal diversity gradient (LDGs) patterns, where diversity is highest at equatorial regions and decreases poleward [20]. Global drinking water viral communities were associated with the use of residual disinfectant. Chlorine has been shown to effectively reduce viral community diversity in drinking water systems [23].

To thoroughly investigate the global diversity and biogeography of DNA viral communities in the AS systems of WWTPs, this study was conducted to mine and examine the viral communities and functions of 144 AS metagenomes taken from 54 WWTPs in 13 countries. The main objectives of this study were (i) to explore the biogeography and diversity of viral communities and functional genes in AS systems, (ii) to investigate how viral functions and virus–host interactions affect microbes in AS systems, (iii) to gain a comprehensive description of viral-specific regulation mechanisms of biogeochemical cycling in AS. Taken together, our findings were beneficial for expanding the understanding of the global diversity and ecology of AS DNA viruses within a theoretical ecology framework.

## Results

### Overview of recovered viral genomes and protein clusters

**Fig. 1:**
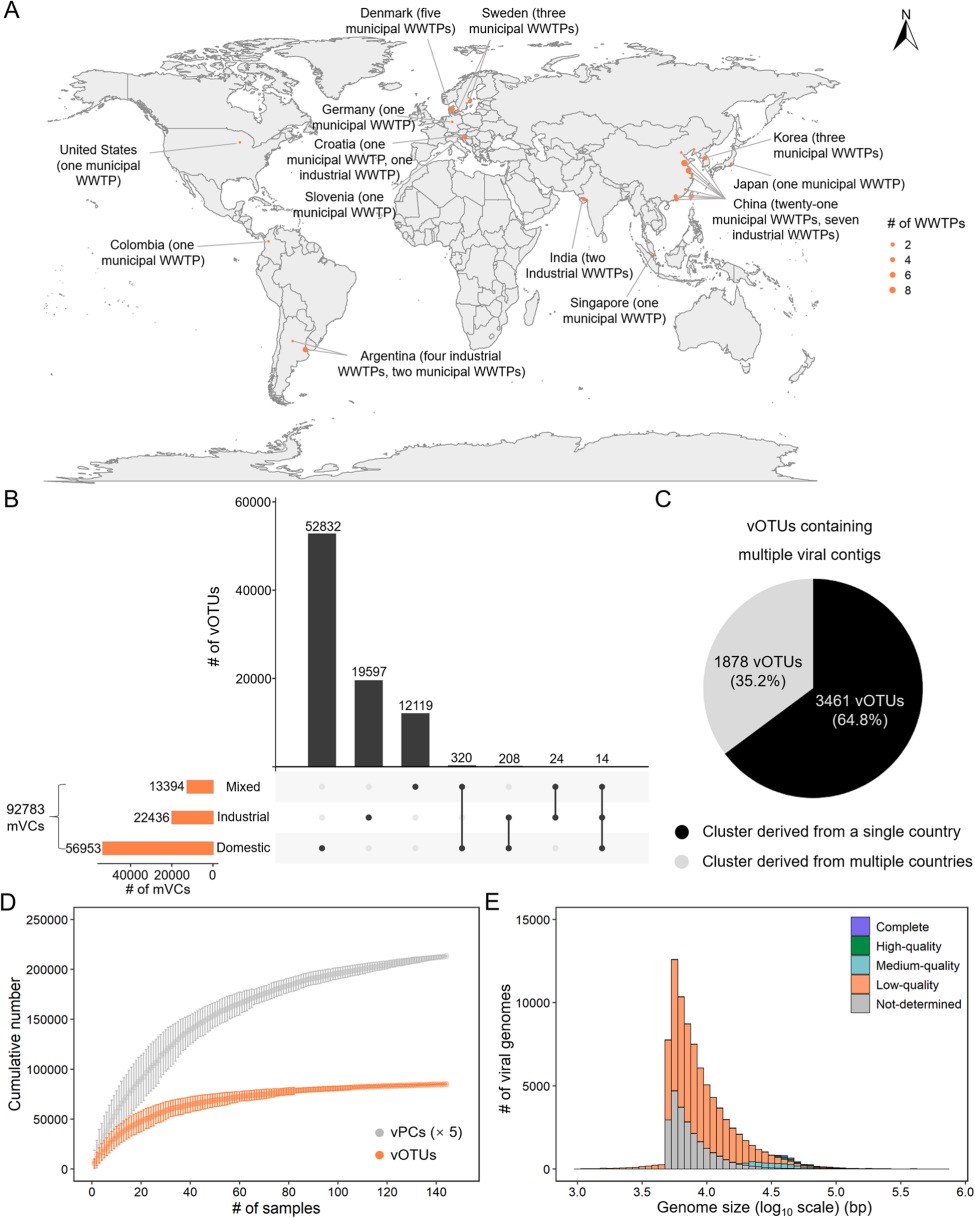
Overview of activated sludge (AS) viruses. **A** Geographic distribution of collected AS samples. The location sites of AS are presented as orange circles, with circle size representing the number of wastewater treatment plants (WWTPs). **B** UpSet plot showing the number of viral operational taxonomic units (vOTUs) and their sharedness between AS with different wastewater types at the species level. **C** Pie chart showing the number of vOTUs clustered in AS from different countries at the species level. **D** Accumulation curve of vOTUs (orange) and viral protein clusters (vPCs, gray). Dots represent the average number of vOTUs and PCs, and error bars represent the range. The numbers of vPCs are divided by five for better visualization. **E** Histogram showing the distribution of viral genome size (log_10_ scale) and quality

The study dataset contained 144 AS metagenome samples from 54 WWTPs (14 industrial WWTPs and 40 municipal WWTPs) in 13 countries, with ~ 1.7 TB sequencing data (Fig. 1A). Depending on the wastewater types, the samples were divided into domestic AS (*n* = 51), industrial AS (*n* = 49), and mixed AS (*n* = 44) (Additional file 2: Table S1). 92,783 metagenomic viral contigs (mVCs) were recovered based on various viral identification methods and further clustered at species-level with 95% average nucleotide identity, yielding 85,114 viral operational taxonomic units (vOTUs) for downstream analysis. In viral genome clustering, mVCs mostly clustered by wastewater type (Fig. 1B). Only 14 vOTUs included mVCs that were present in AS of all three wastewater types, indicating that wastewater type was an important selection factor for viral genomes (Fig. 1B). In addition, 5339 out of 85,114 vOTUs contained multiple mVCs, and 1878 (~ 35.2%) of this subset were detected in more than one country (Fig. 1C).

1,326,402 viral encoded proteins were clustered at 60% identity and 80% coverage, generating 1,115,185 viral protein clusters (vPCs) for viral functional gene analysis. The vOTU and vPC rarefaction curve analyses showed that the amount of sequencing data appeared to be sufficient for detecting the dominant viral members and functional genes in AS (Fig. 1D). In our viral dataset, 80 vOTUs with genomes larger than 200 kb were detected, and likely belonged to potential huge viruses (Fig. 1E) [24]. A total of 1181 vOTUs exhibited genome completeness > 90% with an average size of 55 kb (Fig. 1E). Since AS metagenomes in this study were not specifically enriched for viral particles, most of vOTUs (~ 68.2%) were genomic fragments with the genome completeness < 50% (Fig. 1E). In addition, approximately 27.2% of the vOTUs exhibited no similarity to viruses in CheckV database, which may be the potential novel viruses (Fig. 1E). Spearman’s correlation analysis showed that viral genome size did not correlate with normalized abundance (*P* > 0.05). However, a larger viral genome size indicated higher viral quality (Student’s *t* test, *P* < 0.05).

### Biogeographic patterns and drivers of viral communities

**Fig. 2:**
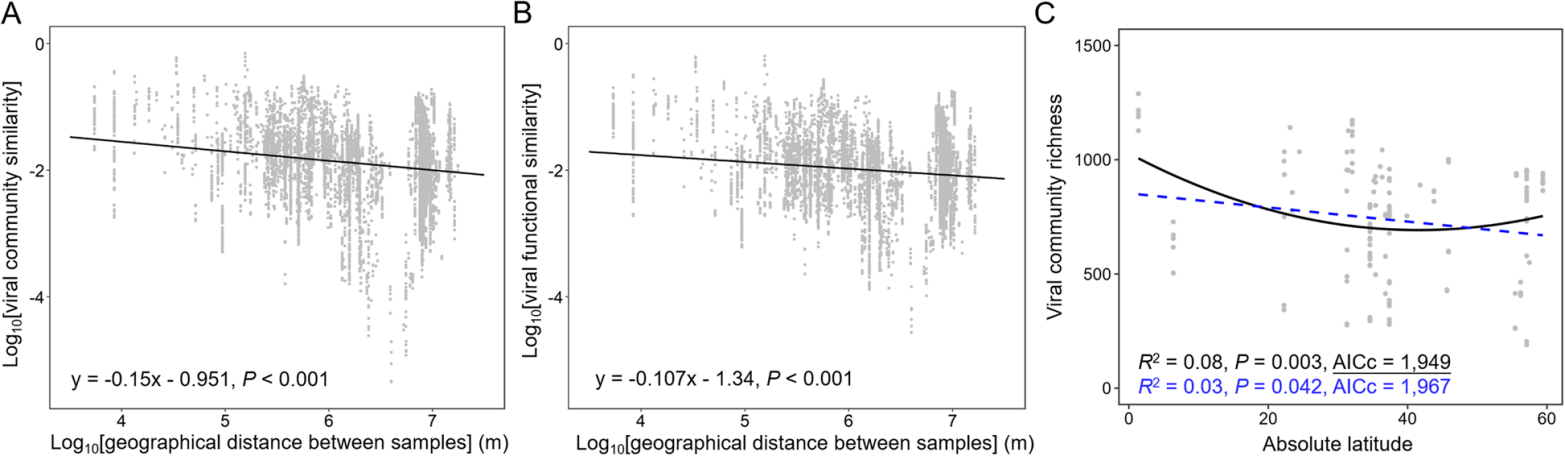
Biogeographic diversity patterns of activated sludge (AS) viruses. **A, B** Distance-decay relationships (DDRs) based on Bray–Curtis dissimilarity of viral community and functional gene structures. **C** Latitudinal diversity patterns of viral community richness. First- and second-order polynomial fits are shown in blue and black, respectively. The best polynomial fit was determined (as underlined) based on the corrected Akaike Information Criterion (AICc)

Two typical and universal biogeographic patterns of macro- and microorganisms, distance-decay relationship (DDR) and latitudinal diversity gradient (LDG) [25–27], were explored in AS viral communities. The results revealed that both viral communities and their functional genes had significant DDR patterns (*P* < 0.001) (Fig. 2A, B). Steeper DDR patterns were observed for viral communities (*S* =  − 0.15) than that for viral functional genes (*S* =  − 0.107) in AS (Fig. 2A, B), indicating that viral functional gene structures were more stable than viral communities at global scale. What is more, typical latitudinal diversity gradient (LDG) pattern was not observed in AS viral communities at a global scale (Fig. 2C). Contrary to AS microbial communities [2], AS viral community diversity was highest at the equator, and decreased towards mid-latitude. And then, it slightly increased from mid-latitude to high-latitude (Fig. 2C).

**Fig. 3:**
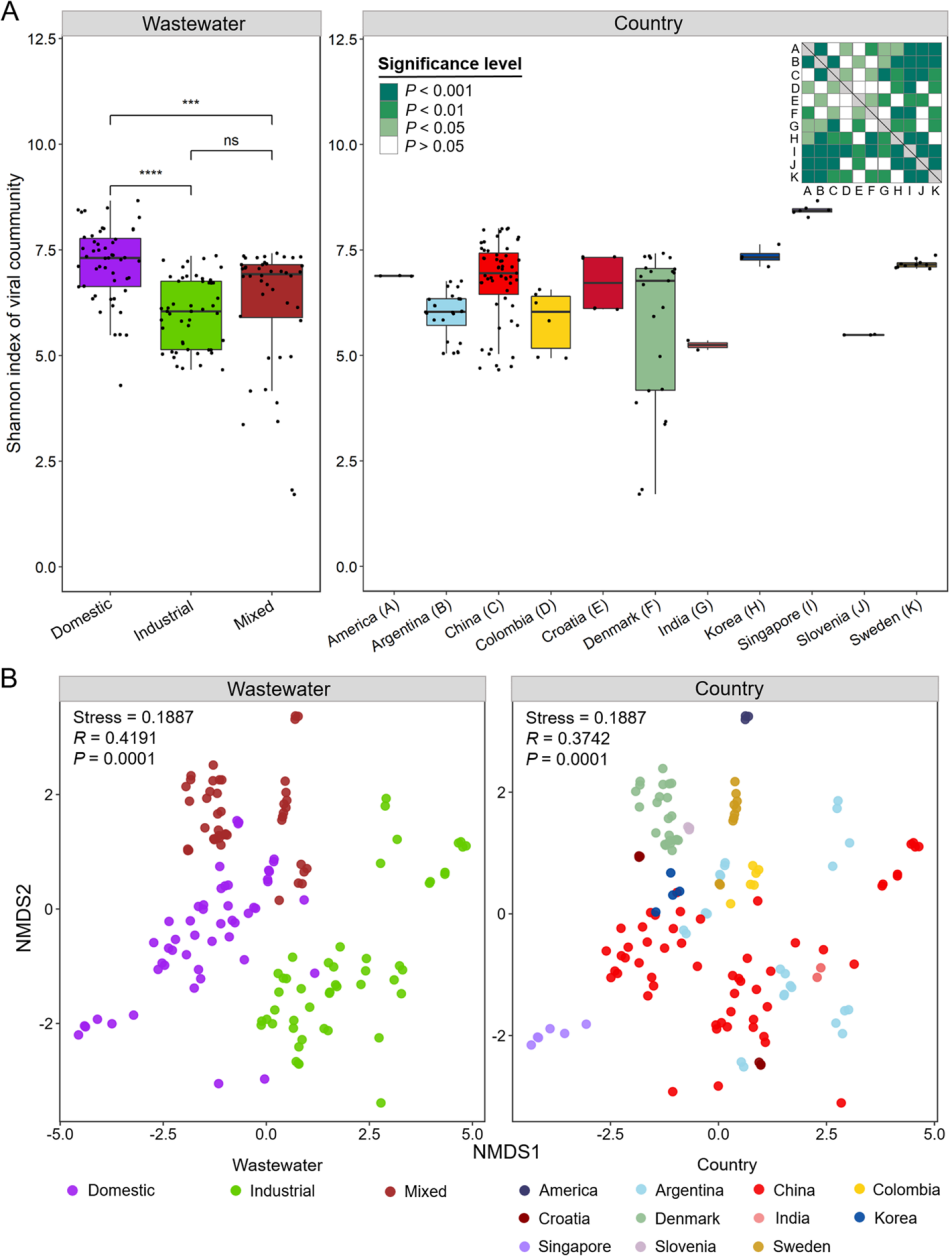
Alpha and beta diversity analysis of AS viral communities. **A** Shannon index of viral communities across different datasets. The significant difference test is determined using Student’s *t*-test, ns indicates no significant difference, *** indicates *P* < 0.001, **** indicates *P* < 0.0001. The significant differences in viral diversity in AS with different countries are shown in the heatmap. **B** NMDS analysis of viral communities based on the Bray–Curtis dissimilarity calculated by the normalized mean coverage of viral operational taxonomic units (vOTUs). ANOSIM is applied to detect the differences in viral communities in AS between different wastewater types or countries

Here, we further analyzed the effects of some abiotic factors, such as wastewater types and geographic factors, on AS viral communities. Alpha diversity analysis was performed based on multiple ecological indices, including Richness, Shannon index, and Simpson index (Additional file 3: Table S2). The results showed that viral community diversity was significantly higher in AS with domestic wastewater than in AS with industrial and mixed wastewaters (Student’s *t* test, *P* < 0.001) (Fig. 3A and Additional file 1: Figure S1). In addition, the viral community diversity was significantly different between various countries, reflecting the effects of geographic factors (Fig. 3A). Beta diversity analysis using nonmetric multidimensional scaling (NMDS) showed that the composition of viral communities was also significantly different between different wastewater types and countries (ANOSIM, *P* = 0.0001) (Fig. 3B).

To assess the relative importance of the geographic factors and wastewater types on the alpha and beta diversity of viral communities, the linear mixed-effects model (LMM) analysis and permutational multivariate analysis of variance (PERMANOVA) were further conducted. As a result, LMM analysis suggested that the geographic factors (*R*^2^ = 0.246, *P* < 0.01) were more critical for causing differences in AS viral community diversity compared to wastewater types (*R*^2^ = 0.036, *P* < 0.05). PERMANOVA indicated that the geographic factors (*R*^2^ = 0.272, *P* = 0.0001) also had more substantial influence than wastewater types (*R*^2^ = 0.081, *P* = 0.0001) on the variations of viral community composition (i.e., beta diversity). Overall, these results revealed the relatively greater importance of the geographic factors in shaping viral community structures than wastewater types, which were consistent with previous findings in AS bacterial communities [2].

### Clustering and taxonomy assignment of viruses

**Fig. 4:**
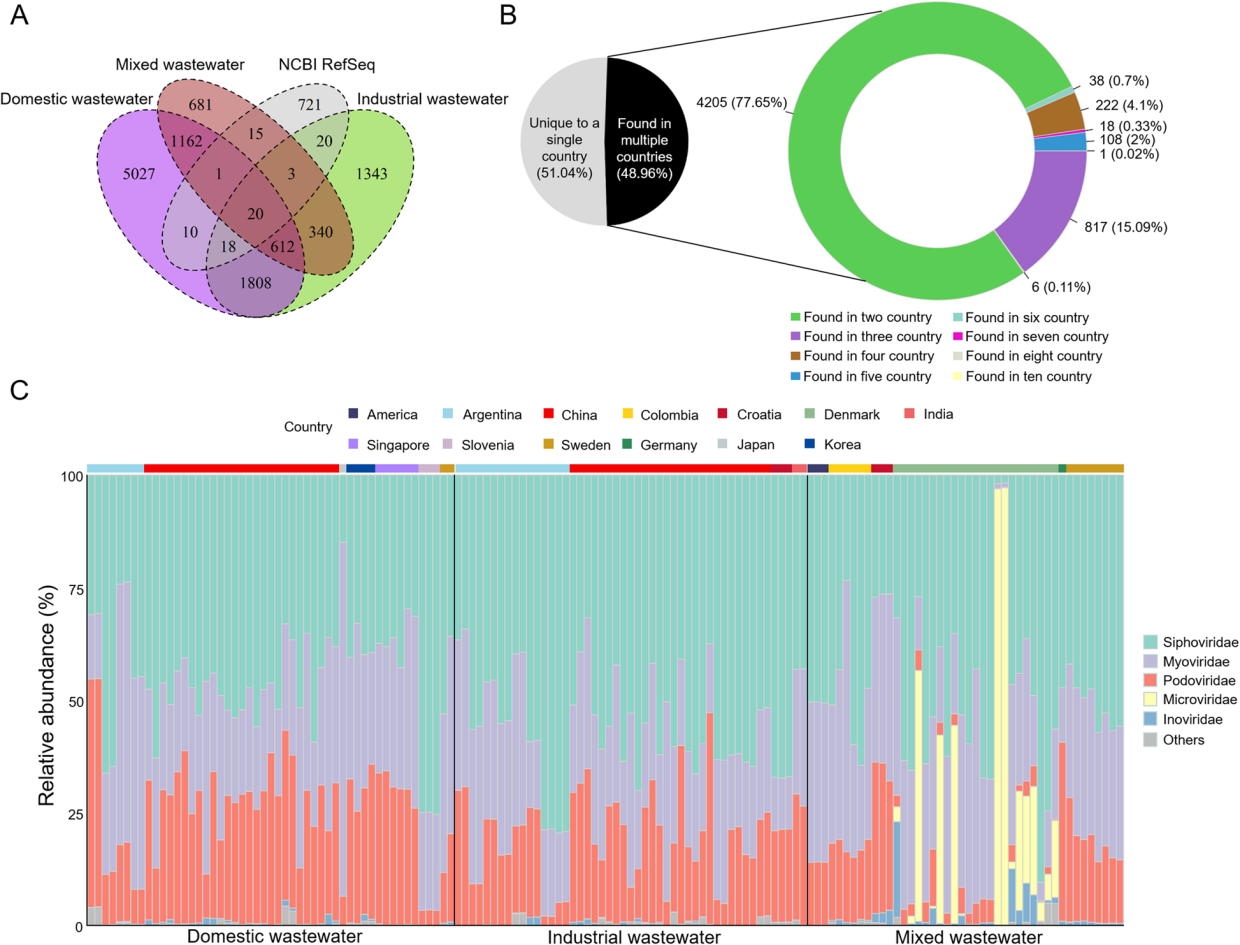
Taxonomy and clustering of activated sludge (AS) viruses with known viruses. **A** The number of shared viral clusters (VCs) in AS with different wastewater types and NCBI viral RefSeq. **B** The number of shared VCs in AS from different countries.** C** The relative abundance of viral taxa (*n* = 22,673) across different AS samples based on the normalized mean coverage. AS samples are separated by wastewater types (black lines) and countries (different colors). Viral taxa with relative abundance of less than 0.1% are classified as others

Clustering network analysis was performed with representative genomes of the 85,114 vOTUs based on viral shared protein clusters. Of the 85,114 vOTUs, 34,967 (~ 41.1%) could be clustered, resulting in 11,060 viral clusters (VCs) (Fig. 4A). Among them, only 482 vOTUs clustered with known viruses (NCBI RefSeq database), generating 87 VCs (Fig. 4A). 632 VCs contained viruses from all three wastewater types (Fig. 4A). Clustering of viruses from AS in different countries showed that 5415 out of 11,060 VCs (~ 48.96%) contained viruses from more than two countries (Fig. 4B). Among these VCs, VC_786 contained 58 vOTUs from ten countries and this cluster had no association with any known viral genome (Additional file 1: Figure S2A), and may be an important, but unexplored VC in AS systems.

22,673 of the 85,114 vOTUs (~ 26.64%) were classified by linking viral proteins to known viruses in the NCBI RefSeq database (Additional file 4: Table S3). 22,463 out of these classified vOTUs belonged to three families (*Siphoviridae*, *Myoviridae*, and *Podoviridae*) of the order *Caudovirales* (Additional file 1: Figure S2B). The relative abundance of *Caudovirales* was dominant in most AS samples, with only AS in Denmark having a high abundance of viruses belonging to the families *Microviridae* and *Inoviridae* (Fig. 4C). Notably, the distribution patterns of viral taxonomy in AS systems were similar within the same region rather than the same wastewater type, suggesting that distance effect was a more important factor in driving viral taxonomy (Fig. 4C). This result was also supported by LMMs (Additional file 5: Table S4). Geographic factors exhibited more significant effects on three major viral families (*Siphoviridae*, *Myoviridae*, and *Podoviridae*) compared to wastewater type (Additional file 5: Table S4).

Considering the high abundance of *Caudovirales* viruses in AS, a phylogenetic analysis of 2612 vOTUs and 1433 viral genomes from RefSeq was further performed using 77 maker genes prevalent in this order (Additional file 1: Figure S3 and Additional file 4: Table S3). Most of these viruses with sufficient marker genes had high genome quality, with the majority belonging to the family *Siphoviridae* (Additional file 1: Figure S3). As previously noted [28], the correspondence between viral classification based on tail morphology and genome-based phylogeny is poor, but these near-complete viral genomes expanded the limited number of *Caudovirales* from AS (Additional file 1: Figure S3).

### Analysis of virus-carried antibiotic resistance genes (ARGs)

**Fig. 5:**
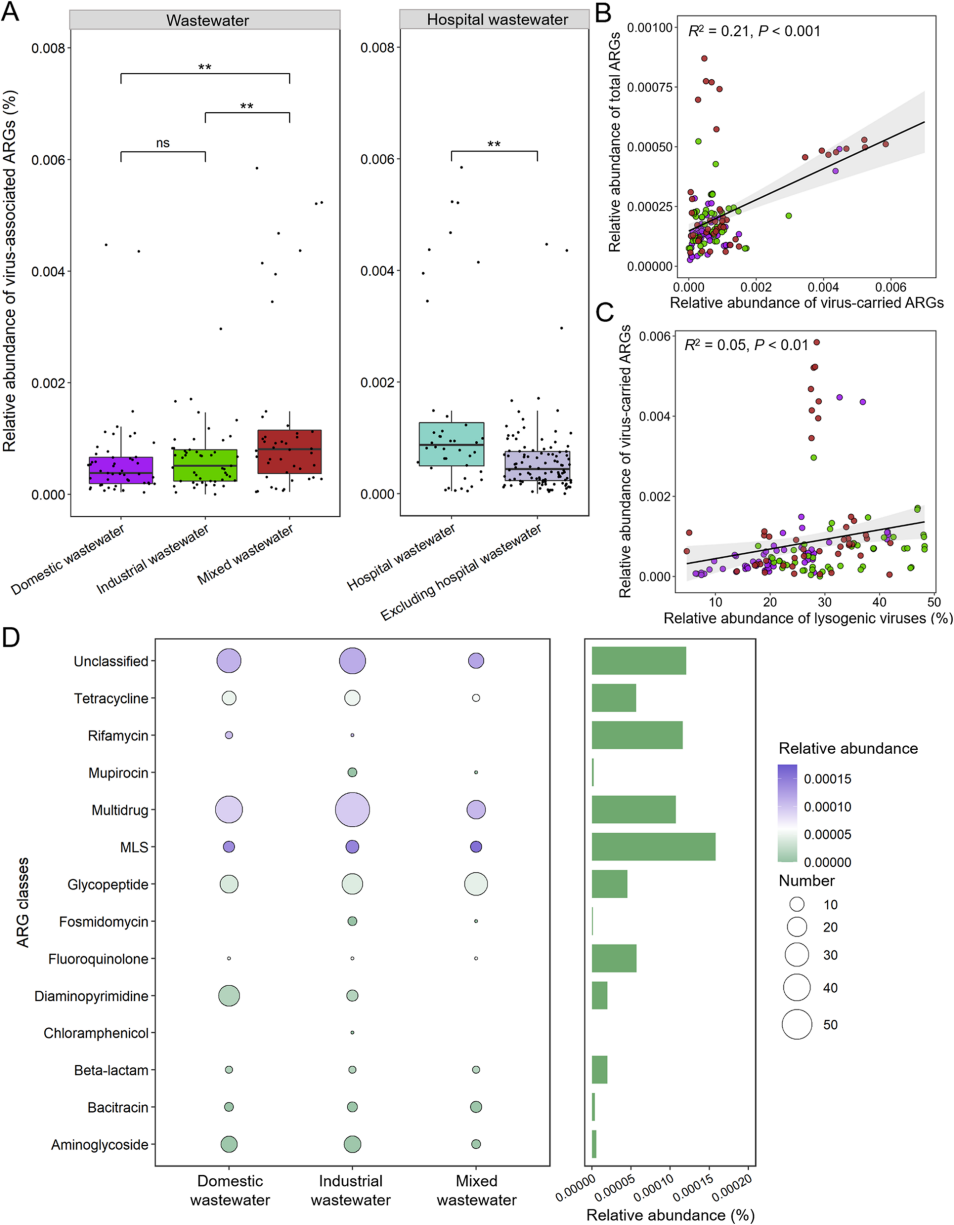
Analysis of antibiotic resistance genes (ARGs) carried by viruses in activated sludge (AS). **A** The relative abundance of ARGs carried by viruses across different AS samples. The significant difference test was determined by using Student’s *t*-test, ns indicates no significant difference, ** indicates *P* < 0.01. **B** Correlation analysis of the relative abundance of virus-associated ARGs with the relative abundance of total ARGs. **C** Correlation analysis of the relative abundance of virus-associated ARGs with the relative abundance of lysogenic viruses. **D** The number and relative abundance distribution of different ARGs classes across AS with the three wastewater types. Bubble size represents the number of ARGs, and the different colors represent the relative abundance of ARGs. The bars represent the total relative abundance of each ARG class

A total of 261 vPCs containing 355 viral genes from 306 vOTUs (~ 0.36% of total vOTUs) were identified as ARGs (Additional file 6: Table S5). Analysis of the relative abundance of virus-carried ARGs showed that viruses carried ARGs more frequently in AS receiving mixed wastewater as compared to AS with domestic and industrial wastewater (Student’s *t* test,* P* < 0.01) (Fig. 5A). Through source tracking of the wastewater receiving source for AS, 40 AS samples were determined to receive hospital wastewater (Additional file 7: Table S6). The relative abundance of virus-carried ARGs was significantly higher in AS receiving hospital wastewater (Student’s *t* test, *P* < 0.01) (Fig. 5A). Owing to hospital wastewater being categorized as mixed wastewater (Additional file 7: Table S6), this may be a major factor behind the high abundance of virus-carried ARGs in AS receiving mixed wastewater.

To explore the contribution of virus-carried ARGs in AS systems, we further performed correlation analysis between the relative abundance of total ARGs with the relative abundance of virus-carried ARGs across each sample (Fig. 5B). As a result, a significant positive correlation was observed (Fig. 5B), suggesting that virus-carried ARGs had contributed to the abundance of environmental ARGs. Notably, the higher abundance of lysogenic viruses in AS also promoted viral carriage of ARGs (Fig. 5C), whereas the abundance of lytic viruses was negatively correlated with the abundance of ARGs carried by viruses (Additional file 1: Figure S4). This result suggested that the lifestyles of viruses (i.e., lysogenic or lytic) were critical for the probability of virus-carried ARGs.

Specifically, virus-associated ARGs in AS covered 13 classes, as well as many unclassified ARGs (Fig. 5D). Among these, the multidrug class was the largest (105 out of 355 ARGs) (Additional file 6: Table S5), while the relative abundance of the macrolide-lincosamide-streptogramin B (MLS) class was the highest (Fig. 5D and Additional file 6: Table S5). Although the relative abundance of most ARGs from the same class varied in AS by wastewater type (9 out of 13 classes, ANOVA, *P* < 0.05), MLS and multidrug classes were always dominant (Fig. 5D). To quantify the contribution of viruses for the HGT of ARGs, we also identified the potential host sources of virus-carried ARGs by searching against the AS microbial genomes. The results showed that 220 out of 263 (~ 84.3%) virus-carried ARGs can be linked to AS microbes (Additional file 6: Table S5), suggesting that HGT processes may occur within their genomes. The potential hosts covered 20 bacterial phyla, of which the phylum *Actinobacteria* was the most abundant (64 out of 220 ARGs). In conclusion, these findings provided some quantified contribution of viruses for the spread of antibiotic resistance in AS.

### Analysis of viral auxiliary metabolic genes (vAMGs)

Eight thousand nine hundred ninety-seven out of 1,115,185 vPCs (~ 0.81%) were identified as vAMGs, covering 11 metabolism classes (Additional file 8: Table S7). Among them, AS viruses tended to encode AMGs for ‘Metabolism of cofactors and vitamins’ (2872 out of 8997), ‘Amino acid metabolism’ (2349 out of 8997), and ‘Carbohydrate metabolism’ (1715 out of 8997) (Additional file 1: Figure S5A). To investigate the impact of viruses on AS biogeochemical cycling, we examined and refined 1008 vAMGs related to C, N, P, and S (sulfur) metabolism (Additional file 1: Figure S5B and Additional file 8: Table S7).

**Fig. 6:**
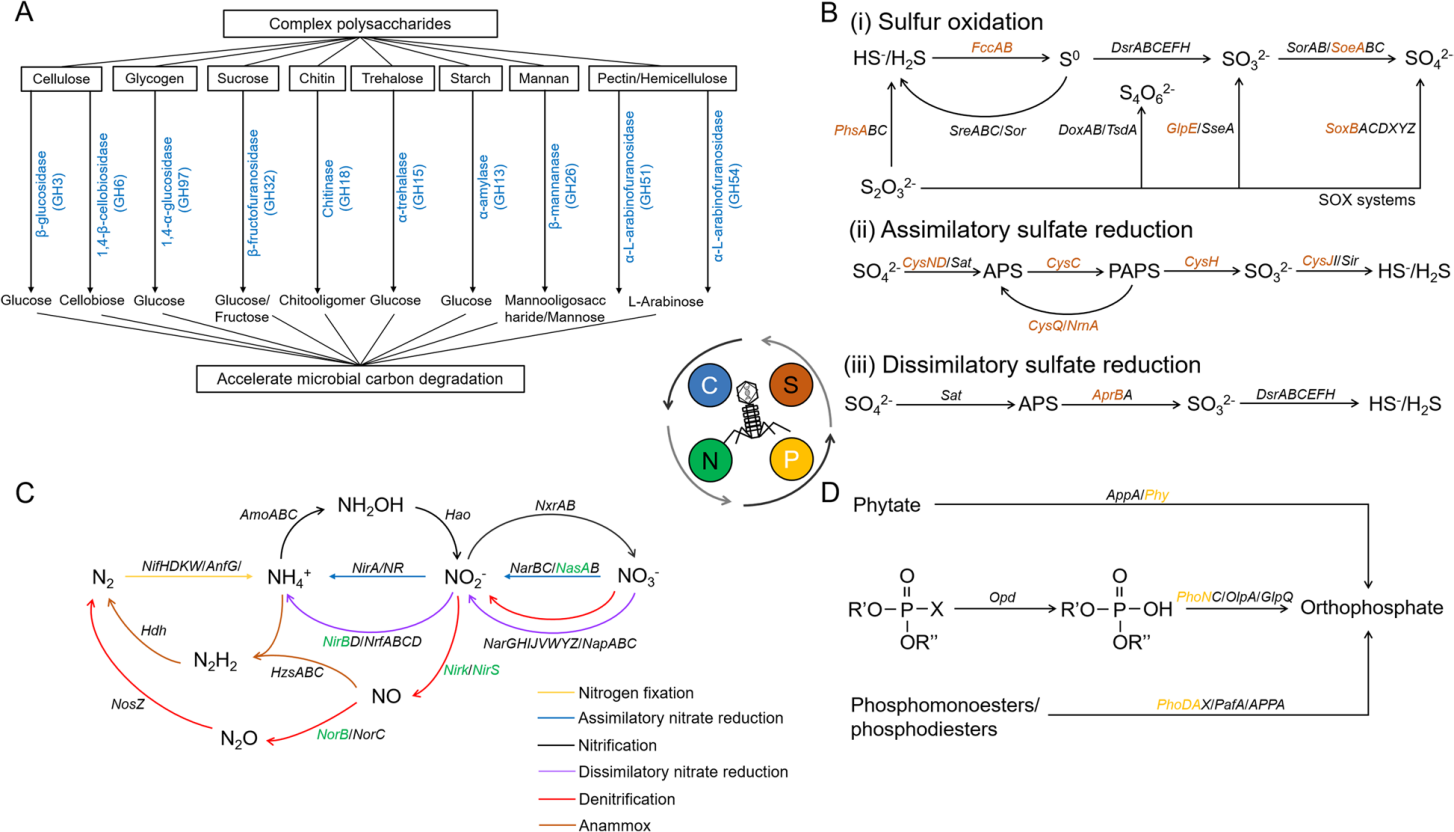
Potential contribution of viruses to biogeochemical cycle in activated sludge (AS). **A** The glycoside hydrolases (GHs) encoded by AS viruses. The schematic shows the degradation of complex polysaccharides by different GHs encoded by AS viruses. The blue font indicates the classes of GHs.** B** Contribution of AS viruses to the sulfur (S) cycle. The schematic shows three pathways of the S cycle, including sulfur oxidation, assimilatory sulfate reduction, and dissimilatory sulfate reduction. Key genes in each pathway are indicated on the arrows with black font. AMGs carried by viruses are colored in brown. **C** Contribution of AS viruses to the nitrogen (N) cycle. The schematic shows the major pathways of the N cycle. The different arrow colors represent different pathways. Key genes in each pathway are indicated on the arrows with black font. AMGs carried by viruses are colored in green. **D** Contribution of AS viruses to the phosphorus (P) cycle. The schematic shows the pathways of organic phosphoester hydrolysis. Key genes in this pathway are indicated on the arrows in black font. AMGs carried by viruses are colored in gold

138 vAMGs were classified as C metabolism genes based on CAZymes database, containing 72 glycoside hydrolases (GHs) of 10 families associated with the degradation of complex polysaccharides (Fig. 6A). For S metabolism, 426 vAMGs were annotated, covering 7 important S metabolism pathways (Fig. 6B). Among them, vAMGs related to assimilatory sulfate reduction (391 out of 426) were the most abundant, including *cysC*/*D*/*N*/*Q*/*J*/*H* and *nrnA* genes, remapping the entire assimilatory sulfate reduction pathway (Fig. 6B and Additional file 9: Table S8). In addition, some viruses encoded *aprB* genes related to dissimilatory sulfate reduction, *phsA* genes related to S reduction, and *fccA*/*B*, *glpE*, *soeA*, and *soxB* genes related to S oxidation (Fig. 6B and Additional file 9: Table S8).

The effective removal of N and P are also among the main objectives of AS systems [29]. The denitrification process plays a key role in biological N removal [29]. We found several vAMGs related to denitrification, including *nirk*, *nirS*, and *norB* genes, demonstrating the contribution of viruses to the biological N removal process (Fig. 6C). In addition, AS virus-encoded *nasA* gene related to assimilatory nitrate reduction, *nirB* gene related to dissimilatory nitrate reduction, and many genes related to organic degradation and synthesis were also detected (Fig. 6C and Additional file 9: Table S8). As emerging contaminants in wastewater, organophosphates pose significant risks to human health [30]. Wastewater treatment is an important method of removing these contaminants, especially the process of organic phosphoester hydrolysis in AS systems. Some key vAMGs involved in the three major organophosphates degradation pathways were identified, including alkaline phosphatase (*phoA* and *phoD*), acid phosphatase (*phoN*) and phytase (*phy*), revealing the critical role of viruses in P metabolism (Fig. 6D).

### Virus-host interaction dynamics

A total of 3823 mOTUs were recovered from the AS samples, including 3777 bacterial and 46 archaeal representative genomes (Additional file 1: Figure S6 and S7). To understand virus-host interactions in AS, 15,353 vOTUs were assigned host information by linking to 3189 host mOTUs (Additional file 10: Table S9). The hosts of these vOTUs covered all archaeal phyla and 46 out of 52 bacterial phyla (Additional file 10: Table S9). The host range of 34 vOTUs spanned multiple phyla, but there were no viruses that could potentially infect both bacteria and archaea (Additional file 10: Table S9). There were 158 vOTUs that could potentially infect archaea, mainly the phyla *Euryarchaeota* (93 vOTUs) and *Thaumarchaeota* (51 vOTUs) (Additional file 1: Figure S6). 15,195 vOTUs could potentially infect bacteria, of which the hosts were dominated by the phylum *Proteobacteria* (5985 out of 15,195 vOTUs) (Additional file 1: Figure S7). Additionally, there were also many viruses that could potentially infect *Bacteroidetes* (2067 vOTUs) and *Actinobacteria* (1539 vOTUs).

**Fig. 7:**
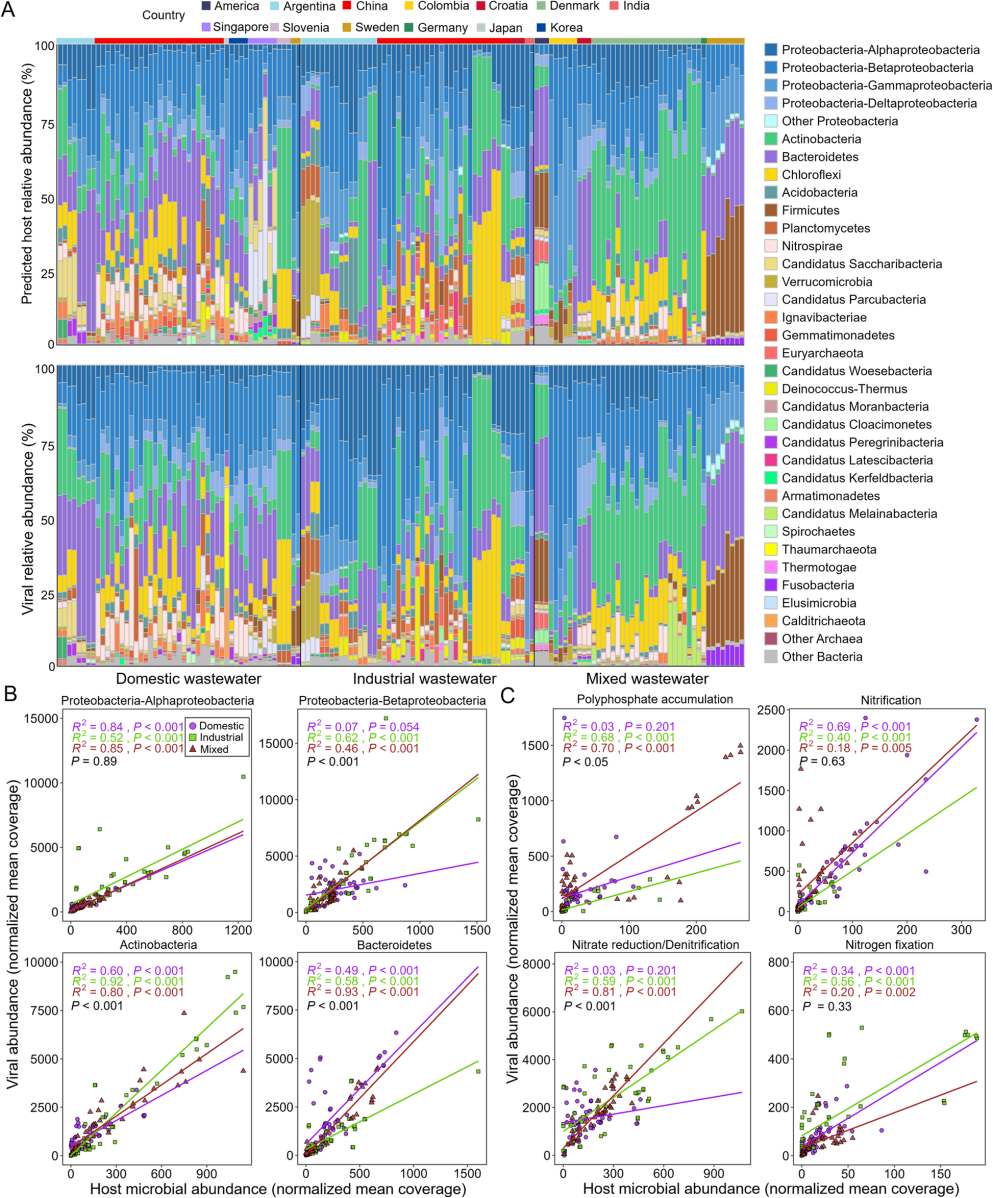
Virus-host interactions in activated sludge (AS). **A** Relative abundances of vOTUs and their hosts grouped by the host taxonomy in each AS ample. AS samples are separated by wastewater types (black lines) and countries (different colors).** B**,** C** Linear regression model analysis is performed based on the virus-host abundance correlations for the specific lineage or functional group in each dataset (domestic wastewater: *n* = 51, industrial wastewater: *n* = 49, and mixed wastewater: *n* = 44). Based on linear regression analysis, the *R*^2^ values and *P* values of each dataset in linear regression models are presented in different colors. Two-way ANOVA *P* values (black font) indicate the significant differences between the three datasets

In general, the normalized abundance showed a strong virus-host correlation (*R*^2^ = 0.95, *P* < 0.001) in AS at the phylum level (class level for *Proteobacteria*) (Additional file 1: Figure S8A and B). The relative abundances of viral communities in each AS sample were highly consistent with that of potential host microbes (Fig. 7A). These results were similar to previous findings in permafrost and marine environments [31, 32], showing that the composition of the host microbial community usually determined the viral community composition and that viruses also regulated host microbial community structure. In the virus-host linkage, the virus/host abundance ratios (VHRs) were greater than one for most specific lineages, revealing the high replication activity of AS viruses (Additional file 1: Figure S9). Among them, the phylum *Candidatus Melainabacteria* had the highest VHR at 19.5 (Additional file 1: Figure S9). Several prokaryotic lineages related to N metabolism in AS also had high VHRs (VHRs > 5), such as the phyla *Nitrospirae* (nitrification) [33], *Thaumarchaeota* (ammonia oxidation) [34], and *Planctomycetes* (anaerobic ammonia oxidation) [35] (Additional file 1: Figure S9).

Two-way ANOVA analysis showed that the virus-host interactions of specific prokaryotic lineages were related to wastewater type (Fig. 7B). For example, the VHRs of the four most abundant prokaryotic lineages in AS exhibited four different patterns: (i) the VHRs of the class *Alphaproteobacteria* were unaffected by wastewater type. (ii) the VHRs of the class *Betaproteobacteria* remained consistent among AS receiving industrial and mixed wastewater, and were significantly higher than those in AS that received domestic wastewater. (iii) the VHRs of the phylum *Actinobacteria* were significantly different for each of the three wastewater types. (iv) The VHRs of the phylum *Bacteroidetes* were similar in AS that received domestic and mixed wastewater, both of which were significantly higher than those of AS receiving industrial wastewater.

We further examined the virus-host interactions of key prokaryotic linages that maintain functions within the AS system (Fig. 7C): (i) For the genera *Pseudomonas*, *Tetrasphaera*, *Aeromonas*, and others associated with biological phosphate elimination, the VHRs of AS that received domestic and industrial wastewater were similar and both were significantly lower than those of AS that received mixed wastewater. (ii) For ammonia-oxidizing bacteria (*Nitrosomonas*), ammonia-oxidizing archaea (*Nitrososphaera*, *Nitrosarchaeum*, etc.), and nitrite oxidation bacteria (*Nitrospira*) with nitrification functions, there were no significant differences in the VHRs between wastewater types. (iii) Nitrate-reducing and denitrifying bacteria mediate the biological N removal in AS, and include the genera *Alicycliphilus*, *Castellaniella*, *Novosphingobium*, etc. The VHRs of these prokaryotic lineages were similar in AS that received industrial and mixed wastewater, while the VHRs in AS that received domestic wastewater were significantly lower. (iv) Nitrogen fixation bacteria in this study included the genera *Rhizobacter*, *Bradyrhizobium*, *Mesorhizobium*, etc. The VHRs of these organisms displayed no differences between the wastewater types. The results demonstrated that viruses can regulate nutrient cycling processes in AS, and that wastewater type significantly influenced viral regulation.

## Discussion

As an artificially managed system with high concentrations of viruses, the viral diversity and functional mechanisms in AS have been poorly explored. Some researchers have turned to high-throughput sequencing of the total viral DNA from AS to resolve DNA viral community diversity [16, 36]. While this approach has expanded our knowledge of prokaryotic DNA viruses in AS, it has been unable to link the abundance of viruses to their prokaryotic hosts. In this context, several studies used high-throughput sequencing of total AS DNA for analysis, elucidating the mechanism of virus-host interactions [15]. However, these studies were regionally limited and hardly represented the global geographic patterns of DNA viruses in AS. Therefore, a comprehensive exploration of AS viruses at a global scale was performed in this study to resolve their underlying diversity and functional mechanisms.

### Global diversity of AS prokaryotic viruses

Here, we recovered 85,114 prokaryotic DNA viruses from 144 AS samples retrieved from 13 countries, expanding the currently limited AS viral database and allowing a deeper understanding of viral communities in AS. By linking the recovered AS viruses to known viruses, the results showed that the majority of viruses in AS are unknown (~ 99.4%) at global scales. However, some unknown viral genomes from different geographic regions had close interconnections, suggesting that core viral groups at the genus level might exist in AS, such as VC_786 which was identified through the viral protein network. *Caudovirales* were the dominant taxonomic groups of the dsDNA viruses in AS, which was consistent with previous findings in marine, soil, and acid mine environments [31, 32, 37], indicating that *Caudovirales* can largely represent the viral taxonomic diversity in AS. Notably, a total of 2612 viruses with sufficient marker genes of *Caudovirales* were mined. Phylogenic analysis of the retrieved *Caudovirales* showed relatively distant affinities with known viruses, further emphasizing the importance of AS viral exploration.

### Biogeography and abiotic drivers of AS viral biodiversity

Revealing the temporal and spatial patterns of microbial communities can contribute to a better understanding of their diversity and potential ecological mechanism [38]. Various fundamental biodiversity patterns have been resolved in macro- and microbial ecology, such as LDG, DDR, taxa-area relationship, and species abundance distribution [39]. Among these, LDG and DDR patterns are the most commonly observed in large-scale ecological analyses. Recent studies have shown that the two patterns are followed by viral communities in some complex natural ecosystems [20, 37], but a systematic study is still lacking in managed ecosystems such as AS system.

The DDR describes the biogeographic distribution pattern where community similarity decreases with increasing geographic distance [40, 41]. Here, we demonstrated the DDR patterns of viral communities and functional genes in AS using the Bray–Curtis dissimilarity, highlighting the importance of heterogeneity and geographic distance in shaping viral biodiversity [25]. Importantly, more significant DDR patterns were observed in AS viral taxonomic groups compared to their functional traits owing to functional redundancy [42]. The LDG illustrates the biogeographic distribution pattern that species richness decreased with increasing absolute latitude [43]. Since AS system is a managed ecosystem under relatively stable and similar conditions everywhere, it may not be anticipated to observe LDG pattern of AS viruses at a global scale [2]. As a result, the decay of viral community richness only occurred from equator to mid-latitude: this paralleled the global ocean virome [20]. Notably, the LDG diversity patterns of AS viral communities were found to be contrary to that of AS bacterial communities [2]. This discrepancy could be primarily attributed to the turnovers of virus-host interactions, specifically, the increased viral abundance leading to the changes in host-microbial abundance [44].

Besides the geographic distance, several abiotic drivers may also significantly influence the viral community structure in AS systems. More specifically, the features of WWTPs that utilized AS process are subject to urban planning of different regions [45, 46]. Thus, different WWTPs use various AS processes to target specific wastewater type [47], such as industrial wastewater. As we expected, both geographic factors and wastewater types significantly AS viral biodiversity. More importantly, the geographic factors divided by different countries exhibited higher importance on the AS viral communities compared to wastewater types, suggesting that the AS viral biodiversity is a critical information on the understanding of public health and urban planning.

### Viruses are key reservoirs of ARGs in AS

Viruses can frequently act as vectors of ARGs in different environments and facilitate the spread of antibiotic resistance by HGT [48, 49]. The high abundance of bacteria and viruses in AS systems provides greater opportunities for HGT to occur [16]. Several studies have focused on the contribution of viruses to the spread of antibiotic resistance in AS, but which factor drove this process has not been determined [15]. In this study, 355 ARGs encompassing 13 categories were identified in AS viruses, emphasizing the importance of viruses as key reservoirs of ARGs. We also found that the abundance of virus-associated ARGs was largely related to the abundance of total ARGs and the proportion of lysogenic viruses, which meant that these two factors collectively drove the probability of ARGs carried by viruses. However, many lysogenic viruses were present as proviruses in host microbial genomes, which cannot be released through spontaneously lysing their hosts. This suggested that high proportion of lysogenic viruses facilitated viruses as key reservoirs of ARGs, but their contribution to the abundance accumulation of total ARGs in AS microbial community cannot be estimated. Importantly, ~ 84.3% virus-carried ARGs can be tracked to potential host microbes, providing some quantified data for the contribution of virus-mediated HGT processes. More strikingly, hospital wastewater, as a major source of ARGs, significantly increased the abundance of ARGs carried by viruses, highlighting the necessity of wastewater treatment according to wastewater source.

### Viruses regulated the nutrient cycling of AS

Even though viruses are ubiquitous in the biosphere, executing critical ecosystem functions and regulating ecosystem stability [50, 51], they are not usually considered to be directly involved in biogeochemical cycling, but can influence important nutrient cycling processes in AS via HGT and host lysis [50]. For example, many AMGs associated with polysaccharide degradation and sulfate reduction were identified in AS viral genomes from Hong Kong and Taiwan [15, 16]. To reveal the functions executed by AS viruses at global scales, we chiefly mined AMGs carried by viruses related to C, N, S, and P metabolism. Similar to previous findings [15], about half of vAMGs had functions associated with polysaccharide degradation and assimilatory sulfate reduction. These AMGs might contribute to viral survival and metabolism in AS, resulting in their being carried by many viruses. In addition, viruses in this study encoded a large number of AMGs related to N and P metabolism, which were mainly involved in denitrification and organic phosphoester hydrolysis, revealing important ways that viruses could regulate biological N and P removal in AS. Through virus-host abundance associations, we identified a number of host microbes with key functions actively infected by AS viruses, including nitrogen fixation bacteria, nitrifying bacteria, nitrate/nitrite reducing bacteria, ammonia-oxidizing archaea, ammonia-oxidizing bacteria, and phosphate accumulating bacteria, emphasizing the importance of viral lysis on AS biogeochemistry. Overall, our results illustrated two specific pathways for viral regulation of nutrient cycling and metabolic processes in AS, providing new insights into AS viral functions.

## Conclusions

In conclusion, our study facilitated the understanding of the biogeography and community diversity of AS viruses at the global scale within an ecological framework. In addition, we described two specific pathways (i.e., viral lysis and viral HGT) by which viruses regulated nutrient cycling in AS systems, highlighting the contribution of viruses to wastewater treatment processes. Finally, we proposed that viruses were key reservoirs of ARGs and can frequently act as HGT vectors to spread ARGs in AS, emphasizing the necessity of studies on viral transduction. Future efforts are needed to link AS environmental factors to viral communities and functions to fully resolve the significance of viral actions on AS pollutant and nutrient removal.

## Methods

### Data acquisition and de novo assembly

Eight AS samples in this study were collected from two WWTPs in Binzhou, Shandong province, China. The samples were centrifuged at 5000 × *g* for 10 min and the supernatant was removed [52, 53]. After grinding the sludge with liquid nitrogen, total DNA was extracted using Omega Soil DNA Kit (Omega Bio-Tek, Norcross, GA, USA). The concentration and purity of extracted DNA was measured with NanoDrop 2000 (Thermo Fisher Scientific, MA, USA). DNA was stored at − 20 °C and used for shotgun sequencing (paired-end, 150 bp) with Illumina NovaSeq 6000 (Illumina Inc., San Diego, CA, USA) using the NovaSeq Reagent Kit according to the manufacturer’s instructions in Shanghai Majorbio Bio-pharm Technology Co., Ltd.

To increase sample size and spatial scale, we also obtained publicly available AS shotgun sequencing samples using the keywords “activated sludge” and “metagenome”. Details of all samples used in this study are listed in Additional file 2: Table S1. In the subsequent analyses, AS samples collected from the same WWTP were considered replicates of each other. Raw reads were first trimmed using Trimmomatic v0.39 (default parameters) [54]. Afterwards, clean reads from each WWTP were co-assembled by MEGAHIT v1.2.9 (default parameters) [55].

### Recovery and taxonomy assignment of metagenome-assembled genomes (MAGs)

Contigs ≥ 1 kb in each assembly set were input to metaWRAP v1.3.2 using binning module (–metabat2 –maxbin2 –concoct) and Bin_refinement module (> 50% completeness and < 10% contamination) to finish binning [56]. The produced bin sets contained 5257 MAGs, and these MAGs were further de-replicated at 95% average nucleotide identity using dRep v3.2.2 [57]. The final dataset included 3823 microbial operational taxonomic units (mOTUs). Taxonomy of representative genomes from the mOTUs was assigned using GTDB-tk v1.7.0 based on the Genome Taxonomy Database R06-RS202 [58, 59]. Then, the classification results were refined by comparing to NCBI taxonomy. Finally, the single-copy genes of representative genomes from the mOTUs identified by GTDB-tk were used to construct the bacterial and archaeal trees by RAxML v8.2.12 [60]. All mOTU information recovered in this study is listed in https://doi.org/10.5281/zenodo.7847962.

### Identification and clustering of viral genomes

Virsorter2 v2.2.3 [61], DeepVirFinder v1.0 [62], VIBRANT v1.2.1 [63], and CheckV v0.8.1 [64] were used to identify and screen viruses from the assembled contigs. For contigs ≥ 5 kb, metagenomic viral contigs (mVCs) were identified using the following criteria: (i) DeepVirFinder score ≥ 0.9 and *P* ≤ 0.01, (ii) high confidence level (score ≥ 0.9 or score ≥ 0.7 but have hallmark genes) of VirSorter2 (–keep-original-sequence), (iii) both identified by DeepVirFinder score ≥ 0.7 and *p* ≤ 0.05 and VirSorter2 score ≥ 0.5. (iv) DeepVirFinder score ≥ 0.7 and *p* ≤ 0.05 and VirSorter2 score ≥ 0.5 were further screened using VIBRANT (virome module).

Then, all identified mVCs were further filtered for proviral regions by CheckV. All mVCs were dereplicated and clustered at 95% average nucleotide identity and 85% alignment fraction of shorter sequences (https://github.com/snayfach/MGV) [28], generating 85,114 vOTUs. Genome completeness assessment of these vOTUs was performed using CheckV. All vOTU information recovered in this study is listed at https://doi.org/10.5281/zenodo.7847962.

### Clustering of viral proteins and taxonomy assignment of vOTUs

Prodigal v2.6.3 (-p meta) was used to predict viral genes [65]. The proteins encoded by viruses were first input to vConTACT2 v0.9.19 (default parameters) to run against the NCBI Viral RefSeq v207 database using DIAMOND (Additional file 3: Table S2) [66–68]. Then, the vOTUs that could not be classified were further annotated using VPF-class at the family level (membership ratio > 0.5, confidence score > 0.5) (Additional file 3: Table S2) [69]. Cytoscape v3.8.0 was used to visualize the viral genes shared network formed by vConTACT2 [70]. All proteins encoded by viruses were clustered using cd-hit v4.8.1 with the parameters set as ‘-c 0.6 -aS 0.8 -n 4 -g 1’ to generate vPCs to represent viral functional structures [8, 37, 71]. A phylogenetic analysis of vOTUs classified as the order *Caudovirales* was performed based on marker genes (be prevalent in more than 10% of the viral genomes and gene copy number less than 1.1) as previously proposed [28, 72]. Finally, the phylogenetic tree was visualized using iTOL [73].

### Identification of antibiotic resistance genes (ARGs) and auxiliary metabolic genes (AMGs) carried by viruses

The vPCs were input to DeepARG v1.0.2 (LS model) to identify and classify ARGs carried by viruses using the following criteria: identity ≥ 40%, coverage ≥ 80%, *e* value < 1e − 10, and probability determined by DeepARG ≥ 0.8 (Additional file 5: Table S4) [74, 75]. The resistance mechanisms of potential ARGs were further annotated by running against the CARD database [76]. Clean reads from each sample were also input to DeepARG (SS model, default parameters) to determine the relative abundance of total ARGs (Additional file 2: Table S1). The vPCs predicted to be ARGs were run against the proteins encoded by mOTUs using DIAMOND v2.0.6 [68] to search host sources with the parameters: identity ≥ 60%, coverage ≥ 80%, *e* value < 1e − 10, bitscore ≥ 50. VIBRANT v1.2.1 [63] was used to identify vAMGs from vPCs (Additional file 6: Table S5). Then, the detailed classifications of genes from CAZymes, N metabolism, P metabolism, and S metabolism-related pathways were performed with CAZymes [77], NCyc [78], PCyc [79], and SCyc [80] databases (Additional file 7: Table S6).

### Host and lifestyle prediction of vOTUs

Three different methods were used to predict hosts of vOTUs: (i) nucleotide sequence homology. Viral genomes were homologously matched to mOTUs based on shared genomic regions through BLASTn [19, 81]. The matches that hit ≥ 2500 bp and had a ≥ 70% identity were retained [19]. (ii) CRISPR spacers matches. CRISPR spacers in mOTUs genomes were extracted by CRT [82] and PILER-CR [83] using a Python script (https://github.com/snayfach/MGV) [28], and run with BLASTn against viral genomes using the following parameters: *e* value ≤ 1e − 5, percentage identity ≥ 95%, and mismatch ≤ 1 [32]. (iii) tRNA matches. tRNA sequences in viral genome were exacted using tRNAscan-SE v2.0.9 [84] and run against mOTUs genomes using BLASTn with the parameters: identity ≥ 90%, coverage ≥ 90%. For each method, only the best match was retained (Additional file 8: Table S7). To determine the lifestyles of viruses (i.e., lytic or lysogenic), the proteins encoded by viruses were run against eggNOG and Pfam database (bit score > 50, and *e* value < 1e − 5) to identify lysogenic signals (including integrase, recombinase, repressor, or provirus) [85, 86].

### Calculating the normalized abundance of vOTUs, mOTUs, and vPCs

For vOTUs and mOTUs, clean reads of each sample were first mapped to representative genomes using BWA-MEM [87] in the Coverm v0.6.1 (https://github.com/wwood/CoverM) pipeline with the parameters: identity ≥ 95% and coverage ≥ 90%. Afterward, the average read depth of each vOTU and mOTU across each sample was calculated by Coverm (‘trimmed_mean’ coverage mode). For vPCs, the genes corresponding to representative proteins were extracted and input to the Coverm pipeline and run with the same parameters as the vOTUs and mOTUs abundance calculation [37]. The average read depth of vOTUs, mOTUs, and vPCs were further normalized as previously described [31, 32]. In brief, the clean reads the number of each sample was divided by one hundred million reads to generate a value ‘A’, and the average reads length were divided by 150 to generate a value ‘B’. Then, the average read depth of each contig/genome/gene was divided by values A and B to obtain normalized abundance.

### Statistical analyses

Data statistics were completed based on numerous packages in R v4.2.0. The Cumulative curves were calculated using the ‘specaccum’ function of vegan package [88]. Pearson/Spearman correlations were performed using the ‘rcorr’ function of Hmisc [89]. Alpha diversity analyses were performed using the vegan package [88]. To eliminate the differences in regional pool, the vOTU table used for calculating LDG patterns was normalized by rarefying to the same sequencing depth using ‘rrarefy’ function of vegan package [88]s. The goodness of fit estimates between first and second-order polynomial models was compared using the corrected Akaike Information Criterion (AICc) calculated by nlme package [90]. Bray–Curtis and Euclidean distances were calculated using the ‘vegdist’ function of vegan [88]. ANOSIM was used to test the significance presented in NMDS. LMMs were performed using the lme4 package [91]. In LMMs, wastewater (inflow) types and geographic factors (country) were used as fixed effects and the assembly quality (N50 metric) was used as a random intercept effect to explore their relative importance on the alpha diversity of viral communities and the taxonomy of viruses. Analysis of variance (ANOVA) was used to test the *p* values and the marginal *R*^2^ (the variance of each fixed effect) from the LMMs was calculated by the partR2 package [92]. The geographic distances between different sites were calculated using the ‘geoXY’ function of SoDA package [37, 93]. The lineage-specific virus-host abundance module for each phylum/class was compared using two-way ANOVA [32].

### Supplementary Information

**Additional file 1:** **Figure S1.** The richness and simpson index of AS viruses. Different alpha diversity indices of viral communities across different datasets. The significant difference test is determined using Student’s *t*-test, * indicates* P* < 0.05, *** indicates *P* < 0.001, **** indicates *P* < 0.0001. **Figure S2.** A core cluster and taxonomic diversity of AS viruses. A A core viral cluster (VC_786) containing 58 vOTUs from ten countries. B The relative abundance of different viral families in classified vOTUs. Nodes represent different viruses and edges represent shared protein cluster content. Different colours represent different countries. Different shapes represent different wastewater types. **Figure S3.** Phylogenetic tree of *Caudovirales* viruses in AS using 77 maker genes. **Figure S4.** Correlation analysis of the relative abundance of virus-associated ARGs with the relative abundance of lytic viruses. **Figure S5.** AMGs encoded by AS viruses. A The number of AMGs in 11 metabolism classes. B The number of AMGs in carbohydrate metabolism, nitrogen metabolism, phosphorus metabolism and sulfur metabolism. **Figure S6.** Phylogenetic tree of predicted archaeal hosts of vOTUs. The number in the color coded column represents the number of viruses which could infect the corresponding archaea taxa. **Figure S7.** Phylogenetic tree of predicted bacterial hosts of vOTUs. The number in parentheses represents the number of viruses which can infect the corresponding bacterial taxon. Phyla with a relative abundance of less than 0.1% are categorized as Other Bacteria, while only those with a relative abundance greater than 0.1% are displayed. **Figure S8.** Host-linked viral abundance in AS. A Correlation analysis between the abundance (normalized mean coverage) of viral operational taxonomic units (vOTUs) and their predicted prokaryotic hosts. The gray shaded area shows 95% confidence interval of the fit. Different color represents various host phyla. B Relative abundances (%) of vOTUs and their predicted prokaryotic hosts grouped by the host taxonomy in AS. Each host phylum is represented by a different color. **Figure S9.** Virus/host abundance ratio and host relative abundance for all predicted hosts.

**Additional file 2:** **Table S1.** Activated sludge (AS) sample information.

**Additional file 3:** **Table S2.** Diversity indices and drivers of activated sludge (AS) samples.

**Additional file 4:** **Table S3.** Taxonomy assignment of viral operational taxonomic units (vOTUs).

**Additional file 5:** **Table S4.** Linear mixed-effects model analysis.

**Additional file 6:** **Table S5.** Virus-associated antibiotic resistance genes (ARGs).

**Additional file 7:** **Table S6.** Antibiotic resistance gene (ARG) information of AS samples.

**Additional file 8:** **Table S7.** Viral auxiliary metabolic gene (AMG) information.

**Additional file 9:** **Table S8.** Viral auxiliary metabolic genes (AMGs) for carbon, nitrogen, phosphorus, and sulfur metabolism.

**Additional file 10:** **Table S9.** Host information of viral operational taxonomic units (vOTUs).

## Data Availability

Raw reads of activated sludge metagenomes generated in this study are deposited in NCBI Sequence Read Archive (SRA) database under project ID PRJNA944788 and PRJNA949407. vOTUs, MAGs, and vPCs sequence and table information are available at https://doi.org/10.5281/zenodo.7847962. R code used in this study are publicly available on GitHub at https://github.com/MengzhiJ/Global-diversity-and-biogeography-of-AS-viral-communities.

## References

[1] Ai C, Yan Z, Zhou H (2019). Metagenomic insights into the effects of seasonal temperature variation on the activities of activated sludge. Microorganisms.

[2] Wu L, Ning D, Zhang B (2019). Global diversity and biogeography of bacterial communities in wastewater treatment plants. Nat Microbiol.

[3] van Loosdrecht MC, Brdjanovic D (2014). Anticipating the next century of wastewater treatment. Science.

[4] Dueholm MKD, Nierychlo M, Andersen KS (2022). MiDAS 4: A global catalogue of full-length 16S rRNA gene sequences and taxonomy for studies of bacterial communities in wastewater treatment plants. Nat Commun.

[5] Saunders AM, Albertsen M, Vollertsen J (2016). The activated sludge ecosystem contains a core community of abundant organisms. ISME J.

[6] Zhang T, Shao M-F, Ye L (2012). 454 Pyrosequencing reveals bacterial diversity of activated sludge from 14 sewage treatment plants. ISME J.

[7] Ji M, Liu Z, Sun K (2021). Bacteriophages in water pollution control: Advantages and limitations. Front Environ Sci Eng.

[8] Paez-Espino D, Eloe-Fadrosh EA, Pavlopoulos GA (2016). Uncovering Earth’s virome. Nature.

[9] Suttle CA (2005). Viruses in the sea. Nature.

[10] Mayers KM, Kuhlisch C, Basso JT et al. Grazing on marine viruses and its biogeochemical implications, Mbio. 2023;e01921–01921. 10.1128/mbio.01921-21 PMC9973340 36715508

[11] Jansson JK, Wu R. Soil viral diversity, ecology and climate change. Nat Rev Microbiol. 2022;1–16. 10.1038/s41579-022-00811-z 36352025

[12] Calero-Cáceres W, Balcázar JL (2019). Antibiotic resistance genes in bacteriophages from diverse marine habitats. Sci Total Environ.

[13] Otawa K, Lee SH, Yamazoe A (2007). Abundance, diversity, and dynamics of viruses on microorganisms in activated sludge processes. Microb Ecol.

[14] Zhang J, Gao Q, Zhang Q (2017). Bacteriophage–prokaryote dynamics and interaction within anaerobic digestion processes across time and space. Microbiome.

[15] Shi L-D, Dong X, Liu Z (2022). A mixed blessing of viruses in wastewater treatment plants. Water Res.

[16] Chen Y, Wang Y, Paez-Espino D (2021). Prokaryotic viruses impact functional microorganisms in nutrient removal and carbon cycle in wastewater treatment plants. Nat Commun.

[17] Li X, Cheng Z, Dang C (2021). Metagenomic and viromic data mining reveals viral threats in biologically treated domestic wastewater. Environmental Science and Ecotechnology.

[18] Gu X, Yang Y, Mao F et al. A comparative study of flow cytometry-sorted communities and shotgun viral metagenomics in a Singapore municipal wastewater treatment plant. iMeta. 2022;1:e39. 10.1002/imt2.39 PMC10989988 38868719

[19] Roux S, Brum JR, Dutilh BE (2016). Ecogenomics and potential biogeochemical impacts of globally abundant ocean viruses. Nature.

[20] Gregory AC, Zayed AA, Conceição-Neto N (2019). Marine DNA viral macro-and microdiversity from pole to pole. Cell.

[21] Shkoporov AN, Clooney AG, Sutton TD (2019). The human gut virome is highly diverse, stable, and individual specific. Cell Host Microbe.

[22] Camarillo-Guerrero LF, Almeida A, Rangel-Pineros G (2021). Massive expansion of human gut bacteriophage diversity. Cell.

[23] Hegarty B, Dai Z, Raskin L (2022). A snapshot of the global drinking water virome: Diversity and metabolic potential vary with residual disinfectant use. Water Res.

[24] Al-Shayeb B, Sachdeva R, Chen L-X (2020). Clades of huge phages from across Earth’s ecosystems. Nature.

[25] Zhou J, Ning D (2017). Stochastic community assembly: does it matter in microbial ecology?. Microbiol Mol Biol Rev.

[26] Tu Q, Deng Y, Yan Q (2016). Biogeographic patterns of soil diazotrophic communities across six forests in the North America. Mol Ecol.

[27] Song W, Liu J, Qin W (2022). Functional traits resolve mechanisms governing the assembly and distribution of nitrogen-cycling microbial communities in the global ocean. MBio.

[28] Nayfach S, Páez-Espino D, Call L (2021). Metagenomic compendium of 189,680 DNA viruses from the human gut microbiome. Nat Microbiol.

[29] Lochmatter S, Gonzalez-Gil G, Holliger C (2013). Optimized aeration strategies for nitrogen and phosphorus removal with aerobic granular sludge. Water Res.

[30] Silva M, Baltrusaitis J (2021). Destruction of emerging organophosphate contaminants in wastewater using the heterogeneous iron-based photo-Fenton-like process. J Hazard Mater Lett.

[31] Li Z, Pan D, Wei G (2021). Deep sea sediments associated with cold seeps are a subsurface reservoir of viral diversity. ISME J.

[32] Emerson JB, Roux S, Brum JR (2018). Host-linked soil viral ecology along a permafrost thaw gradient. Nat Microbiol.

[33] Daims H, Nielsen JL, Nielsen PH (2001). In situ characterization of Nitrospira-like nitrite-oxidizing bacteria active in wastewater treatment plants. Appl Environ Microbiol.

[34] Könneke M, Bernhard AE, de La Torre JR (2005). Isolation of an autotrophic ammonia-oxidizing marine archaeon. Nature.

[35] Wiegand S, Jogler M, Jogler C (2018). On the maverick Planctomycetes. FEMS Microbiol Rev.

[36] Wang Y, Jiang X, Liu L (2018). High-resolution temporal and spatial patterns of virome in wastewater treatment systems. Environ Sci Technol.

[37] Gao S, Paez-Espino D, Li J (2022). Patterns and ecological drivers of viral communities in acid mine drainage sediments across Southern China. Nat Commun.

[38] Liu X, Li H, Song W (2023). Distinct ecological mechanisms drive the spatial scaling of abundant and rare microbial taxa in a coastal sediment. J Biogeogr.

[39] Zhou J, Ning D. Stochastic community assembly: does it matter in microbial ecology?. Microbiol Mol Biol Rev. 2017;81. 10.1128/mmbr.00002-00017. 10.1128/MMBR.00002-17 PMC5706748 29021219

[40] Soininen J, McDonald R, Hillebrand H (2007). The distance decay of similarity in ecological communities. Ecography.

[41] Morlon H, Schwilk DW, Bryant JA (2011). Spatial patterns of phylogenetic diversity. Ecol Lett.

[42] Louca S, Polz MF, Mazel F (2018). Function and functional redundancy in microbial systems. Nat Ecol Evol.

[43] Hillebrand H (2004). On the generality of the latitudinal diversity gradient. Am Nat.

[44] Yang K, Wang X, Hou R (2023). Rhizosphere phage communities drive soil suppressiveness to bacterial wilt disease. Microbiome.

[45] Zhao X, Jin X, Guo W (2019). China's urban methane emissions from municipal wastewater treatment plant. Earth's Future.

[46] Zhang Y, Duan L, Wang B (2019). Wastewater-based epidemiology in Beijing, China: prevalence of antibiotic use in flu season and association of pharmaceuticals and personal care products with socioeconomic characteristics. Environ Int.

[47] Zhang Y, Zhang C, Qiu Y (2020). Wastewater treatment technology selection under various influent conditions and effluent standards based on life cycle assessment. Resour Conserv Recycl.

[48] Mirzaei MK, Xue J, Costa R (2021). Challenges of studying the human virome–relevant emerging technologies. Trends Microbiol.

[49] Calero-Cáceres W, Ye M, Balcázar JL (2019). Bacteriophages as environmental reservoirs of antibiotic resistance. Trends Microbiol.

[50] Trubl G, Jang HB, Roux S (2018). Soil viruses are underexplored players in ecosystem carbon processing. MSystems.

[51] Suttle CA (2007). Marine viruses—major players in the global ecosystem. Nat Rev Microbiol.

[52] Bengtsson-Palme J, Milakovic M, Švecová H (2019). Industrial wastewater treatment plant enriches antibiotic resistance genes and alters the structure of microbial communities. Water Res.

[53] Munck C, Albertsen M, Telke A (2015). Limited dissemination of the wastewater treatment plant core resistome. Nat Commun.

[54] Bolger AM, Lohse M, Usadel B (2014). Trimmomatic: a flexible trimmer for Illumina sequence data. Bioinformatics.

[55] Li D, Liu C-M, Luo R (2015). MEGAHIT: an ultra-fast single-node solution for large and complex metagenomics assembly via succinct de Bruijn graph. Bioinformatics.

[56] Uritskiy GV, DiRuggiero J, Taylor J (2018). MetaWRAP—a flexible pipeline for genome-resolved metagenomic data analysis. Microbiome.

[57] Olm MR, Brown CT, Brooks B (2017). dRep: a tool for fast and accurate genomic comparisons that enables improved genome recovery from metagenomes through de-replication. ISME J.

[58] Parks DH, Chuvochina M, Waite DW (2018). A standardized bacterial taxonomy based on genome phylogeny substantially revises the tree of life. Nat Biotechnol.

[59] Chaumeil P-A, Mussig AJ, Hugenholtz P et al. GTDB-Tk: a toolkit to classify genomes with the Genome Taxonomy Database. Oxford University Press; 2020. 10.1093/bioinformatics/btz848 PMC7703759 31730192

[60] Stamatakis A (2014). RAxML version 8: a tool for phylogenetic analysis and post-analysis of large phylogenies. Bioinformatics.

[61] Guo J, Bolduc B, Zayed AA (2021). VirSorter2: a multi-classifier, expert-guided approach to detect diverse DNA and RNA viruses. Microbiome.

[62] Ren J, Song K, Deng C (2020). Identifying viruses from metagenomic data using deep learning. Quant Biol.

[63] Kieft K, Zhou Z, Anantharaman K (2020). VIBRANT: automated recovery, annotation and curation of microbial viruses, and evaluation of viral community function from genomic sequences. Microbiome.

[64] Nayfach S, Camargo AP, Schulz F (2021). CheckV assesses the quality and completeness of metagenome-assembled viral genomes. Nat Biotechnol.

[65] Hyatt D, Chen G-L, LoCascio PF (2010). Prodigal: prokaryotic gene recognition and translation initiation site identification. BMC Bioinformatics.

[66] O'Leary NA, Wright MW, Brister JR (2016). Reference sequence (RefSeq) database at NCBI: current status, taxonomic expansion, and functional annotation. Nucleic Acids Res.

[67] Bin Jang H, Bolduc B, Zablocki O (2019). Taxonomic assignment of uncultivated prokaryotic virus genomes is enabled by gene-sharing networks. Nat Biotechnol.

[68] Buchfink B, Xie C, Huson DH (2015). Fast and sensitive protein alignment using DIAMOND. Nat Methods.

[69] Pons JC, Paez-Espino D, Riera G (2021). VPF-Class: taxonomic assignment and host prediction of uncultivated viruses based on viral protein families. Bioinformatics.

[70] Shannon P, Markiel A, Ozier O (2003). Cytoscape: a software environment for integrated models of biomolecular interaction networks. Genome Res.

[71] Fu L, Niu B, Zhu Z (2012). CD-HIT: accelerated for clustering the next-generation sequencing data. Bioinformatics.

[72] Low SJ, Džunková M, Chaumeil P-A (2019). Evaluation of a concatenated protein phylogeny for classification of tailed double-stranded DNA viruses belonging to the order Caudovirales. Nat Microbiol.

[73] Letunic I, Bork P (2019). Interactive Tree Of Life (iTOL) v4: recent updates and new developments. Nucleic Acids Res.

[74] Arango-Argoty G, Garner E, Pruden A (2018). DeepARG: a deep learning approach for predicting antibiotic resistance genes from metagenomic data. Microbiome.

[75] Chen M-L, An X-L, Liao H (2021). Viral community and virus-associated antibiotic resistance genes in soils amended with organic fertilizers. Environ Sci Technol.

[76] Alcock BP, Raphenya AR, Lau TT (2020). CARD 2020: antibiotic resistome surveillance with the comprehensive antibiotic resistance database. Nucleic Acids Res.

[77] Drula E, Garron M-L, Dogan S (2022). The carbohydrate-active enzyme database: functions and literature. Nucleic Acids Res.

[78] Tu Q, Lin L, Cheng L (2019). NCycDB: a curated integrative database for fast and accurate metagenomic profiling of nitrogen cycling genes. Bioinformatics.

[79] Zeng J, Tu Q, Yu X (2022). PCycDB: a comprehensive and accurate database for fast analysis of phosphorus cycling genes. Microbiome.

[80] Yu X, Zhou J, Song W (2021). SCycDB: a curated functional gene database for metagenomic profiling of sulphur cycling pathways. Mol Ecol Resour.

[81] Camacho C, Coulouris G, Avagyan V (2009). BLAST+: architecture and applications. BMC Bioinformatics.

[82] Bland C, Ramsey TL, Sabree F (2007). CRISPR recognition tool (CRT): a tool for automatic detection of clustered regularly interspaced palindromic repeats. BMC Bioinformatics.

[83] Edgar RC (2007). PILER-CR: fast and accurate identification of CRISPR repeats. BMC Bioinformatics.

[84] Chan PP, Lin BY, Mak AJ et al. tRNAscan-SE 2.0: improved detection and functional classification of transfer RNA genes. Nucleic Acids Res. 2021;49:9077–9096. 10.1093/nar/gkab688 PMC8450103 34417604

[85] Huerta-Cepas J, Szklarczyk D, Heller D et al. eggNOG 5.0: a hierarchical, functionally and phylogenetically annotated orthology resource based on 5090 organisms and 2502 viruses. Nucleic Acids Res. 2019;47:D309-D314. 10.1093/nar/gky1085 PMC6324079 30418610

[86] Mistry J, Chuguransky S, Williams L (2021). Pfam: The protein families database in 2021. Nucleic Acids Res.

[87] Li H. Aligning sequence reads, clone sequences and assembly contigs with BWA-MEM. arXiv preprint arXiv. 1303.3997 2013.

[88] Oksanen J, Blanchet FG, Kindt R et al. Package ‘vegan’, Community ecology package, version. 2013;2:1-295.

[89] Harrell Jr FE, Harrell Jr MFE. Package ‘hmisc’, CRAN2018. 2019;2019:235–236.

[90] Pinheiro J, Bates D, DebRoy S et al. Package ‘nlme’, Linear and nonlinear mixed effects models, version. 2017;3:274.

[91] Bates D, Mächler M, Bolker B et al. Fitting linear mixed-effects models using lme4. arXiv preprint arXiv:1406.5823 2014.

[92] Stoffel MA, Nakagawa S, Schielzeth H (2021). partR2: Partitioning R2 in generalized linear mixed models. PeerJ.

[93] Vincenty T (1975). Direct and inverse solutions of geodesics on the ellipsoid with application of nested equations. Surv Rev.

