# Supplementary material for: Global diversity and biogeography of DNA viral communities in activated sludge systems
Source: Microbiome. 2023 Oct 21;11:234. doi: 10.1186/s40168-023-01672-1 (PMC10589946; doi:10.1186/s40168-023-01672-1)
Supplement: Supplementary file 2 — Additional file 1: Figure S1. The richness and simpson index of AS viruses. Different alpha diversity indices of viral communities across different datasets. The significant difference test is determined using Student’s t-test, * indicates P < 0.05, *** indicates P < 0.001, **** indicates P < 0.0001. Figure S2. A core cluster and taxonomic diversity of AS viruses. A A core viral cluster (VC_786) containing 58 vOTUs from ten countries. B The relative abundance of different viral families in classified vOTUs. Nodes represent different viruses and edges represent shared protein cluster content. Different colours represent different countries. Different shapes represent different wastewater types. Figure S3. Phylogenetic tree of Caudovirales viruses in AS using 77 maker genes. Figure S4. Correlation analysis of the relative abundance of virus-associated ARGs with the relative abundance of lytic viruses. Figure S5. AMGs encoded by AS viruses. A The number of AMGs in 11 metabolism classes. B The number of AMGs in carbohydrate metabolism, nitrogen metabolism, phosphorus metabolism and sulfur metabolism. Figure S6. Phylogenetic tree of predicted archaeal hosts of vOTUs. The number in the color coded column represents the number of viruses which could infect the corresponding archaea taxa. Figure S7. Phylogenetic tree of predicted bacterial hosts of vOTUs. The number in parentheses represents the number of viruses which can infect the corresponding bacterial taxon. Phyla with a relative abundance of less than 0.1% are categorized as Other Bacteria, while only those with a relative abundance greater than 0.1% are displayed. Figure S8. Host-linked viral abundance in AS. A Correlation analysis between the abundance (normalized mean coverage) of viral operational taxonomic units (vOTUs) and their predicted prokaryotic hosts. The gray shaded area shows 95% confidence interval of the fit. Different color represents various host phyla. B Relative abundances (%) of vOTUs and their p [file 40168_2023_1672_MOESM1_ESM.pdf]

# Supporting Information for

## Global diversity and biogeography of DNA viral communities in activated sludge systems

**Author name:** Xiangyu Fan[1,2#*], Mengzhi Ji[1,3#], Dashuai Mu[4,5], Xianghe Zeng[1], Zhen Tian[2], Kaili Sun[1], Rongfeng Gao[1], Yang Liu[2], Xinyuan He[2], Linwei Wu[6*], Qiang Li[1*]

[1]School of Biological Science and Technology, University of Jinan, Jinan, Shandong Province, China

[2]Artificial Intelligence Institute, University of Jinan, Jinan, Shandong Province, China

[3]Institute of Marine Science and Technology, Shandong University, Qingdao, Shandong Province, China

[4]State Key Laboratory of Microbial Technology, Institute of Microbial Technology, Shandong University, Qingdao, Shandong Province, China

[5]Marine College, Shandong University, Weihai, Shandong Province, China

[6]Institute of Ecology, Key Laboratory for Earth Surface Processes of the Ministry of Education, College of Urban and Environmental Sciences, Peking University, Beijing, China

*Correspondence author: fxysnd@126.com (X.F.); linwei.wu@pku.edu.cn (L.W.); chm_liq@ujn.edu.cn (Q.L.)

[#]These authors have contributed equally to this work.

**This PDF file includes:**

Figures S1 to S9

**Other supporting materials for this manuscript include the following:**

Tables S1 to S9

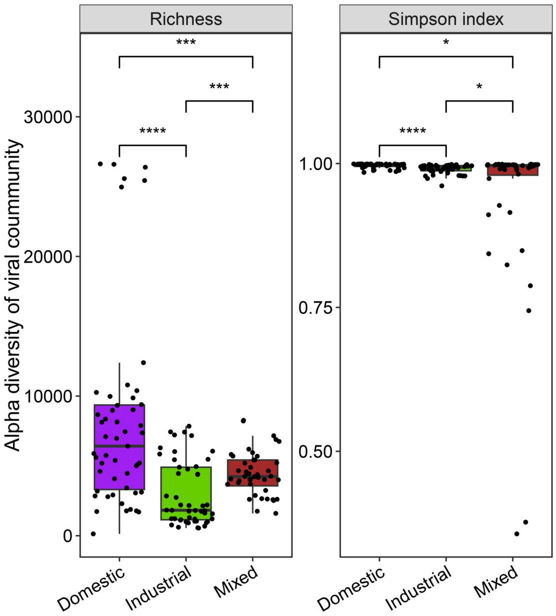


**Fig. S1 The richness and simpson index of AS viruses.** Different alpha diversity indices of viral communities across different datasets. The significant difference test was determined using Student's *t*-test, * indicates $P$ < 0.05, *** indicates $P$ < 0.001, **** indicates $P$ < 0.0001.

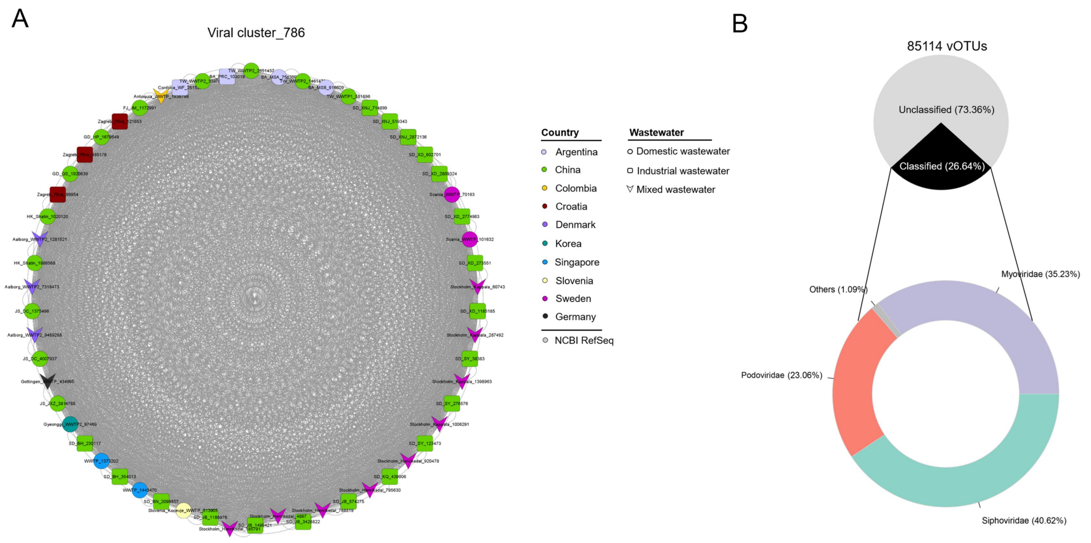


**Fig. S2 A core cluster and taxonomic diversity of AS viruses. A** A core viral cluster (VC_786) containing 58 vOTUs from ten countries. **B** The relative abundance of different viral families in classified vOTUs. Nodes represent different viruses and edges represent shared protein cluster content. Different colours represent different countries. Different shapes represent different wastewater types.

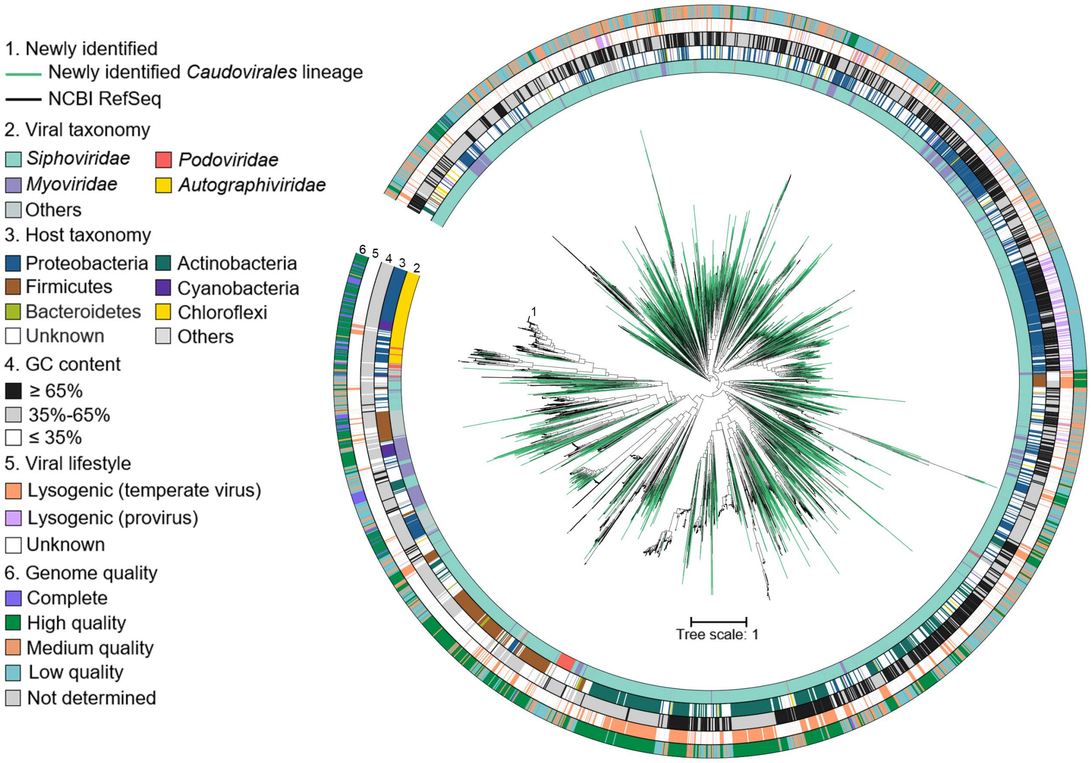


**Fig. S3 Phylogenetic tree of *Caudovirales* viruses in AS using 77 maker genes.**

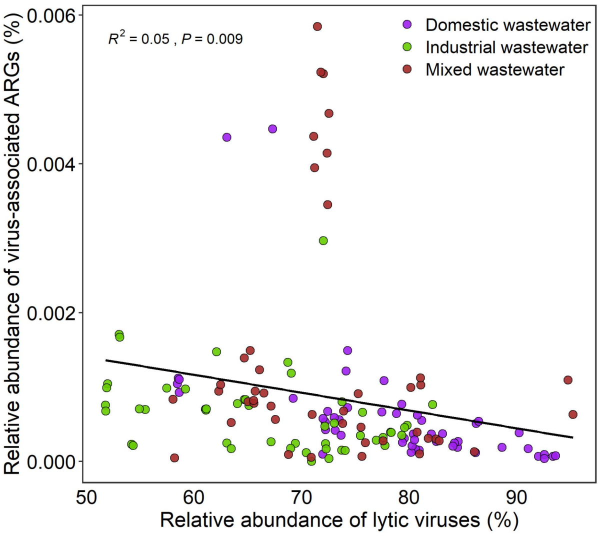


**Fig. S4 Correlation analysis of the relative abundance of virus-associated ARGs with the relative abundance of lytic viruses.**

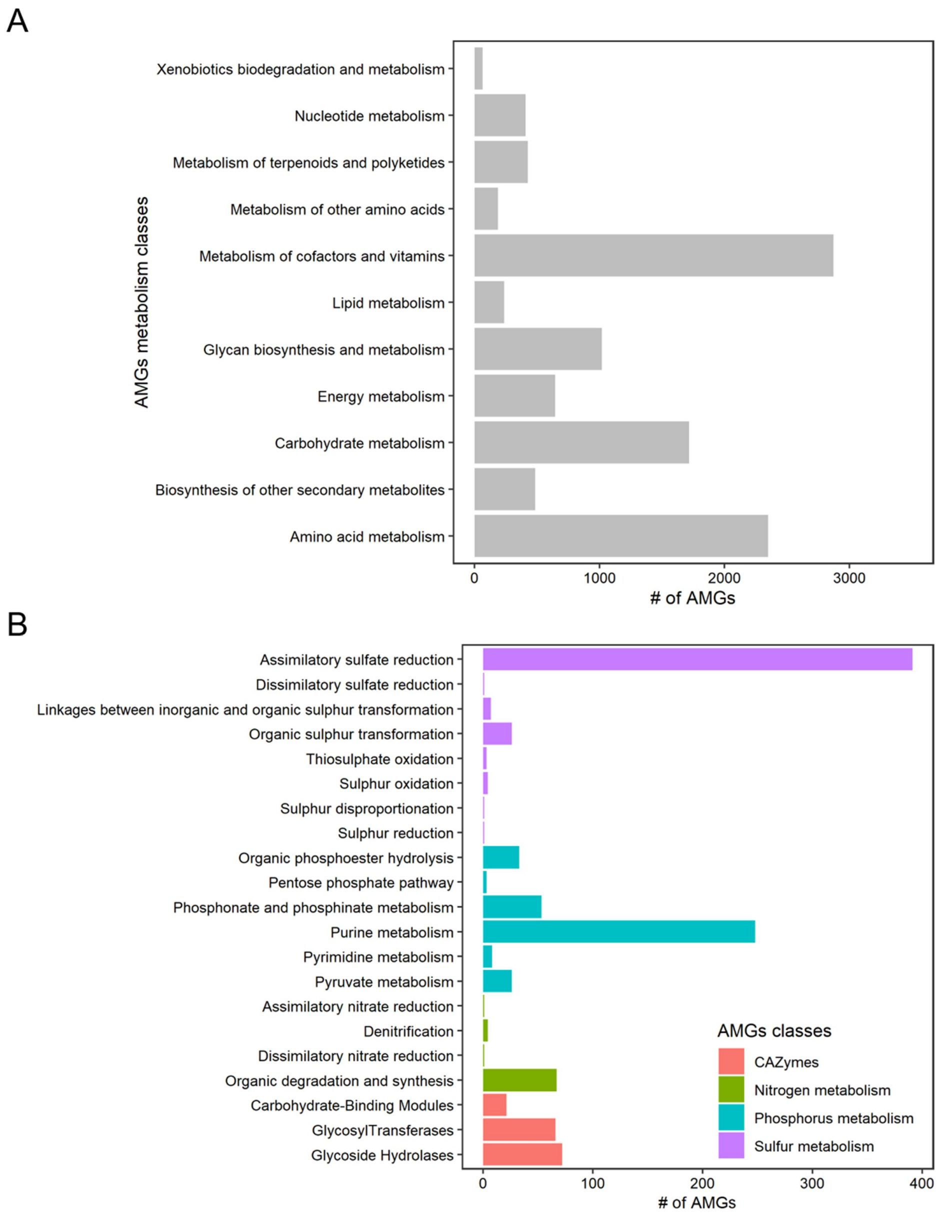


**Fig. S5 AMGs encoded by AS viruses. A** The number of AMGs in 11 metabolism classes. **B** The number of AMGs in carbohydrate metabolism, nitrogen metabolism, phosphorus metabolism and sulfur metabolism.

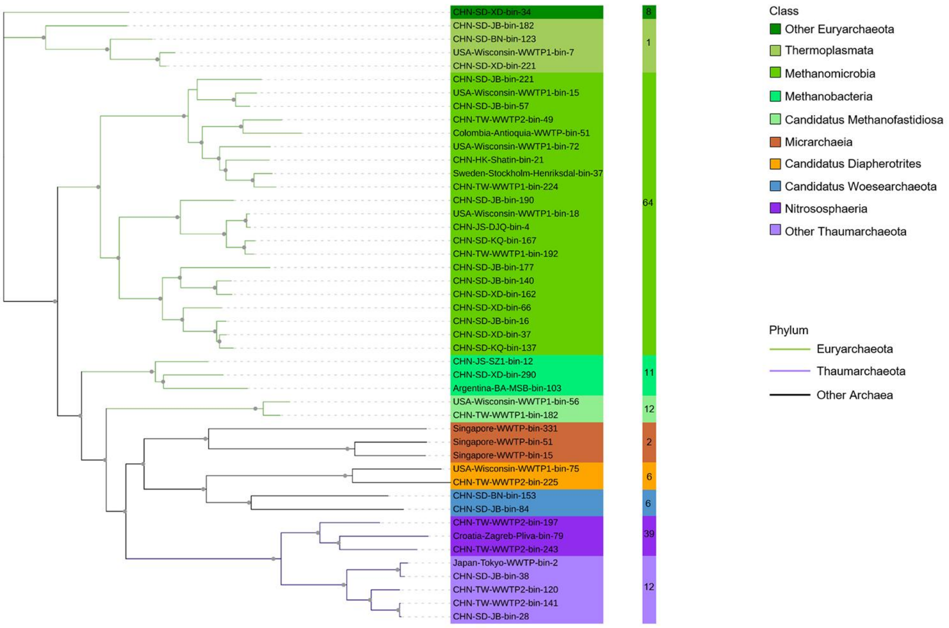


**Fig. S6 Phylogenetic tree of predicted archaeal hosts of vOTUs.** The number in the color coded column represents the number of viruses which could infect the corresponding archaea taxa.

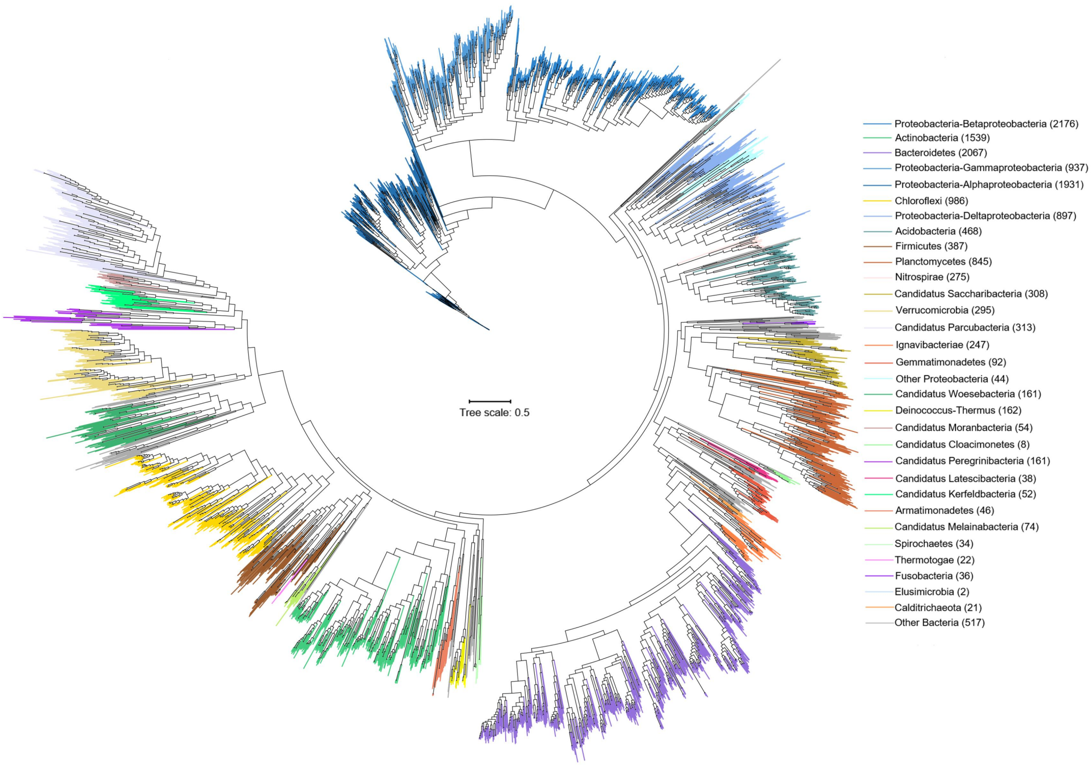


**Fig. S7 Phylogenetic tree of predicted bacterial hosts of vOTUs.** The number in parentheses represents the number of viruses which can infect the corresponding bacterial taxon. Phyla with a relative abundance of less than 0.1% are categorized as Other Bacteria, while only those with a relative abundance greater than 0.1% are displayed.

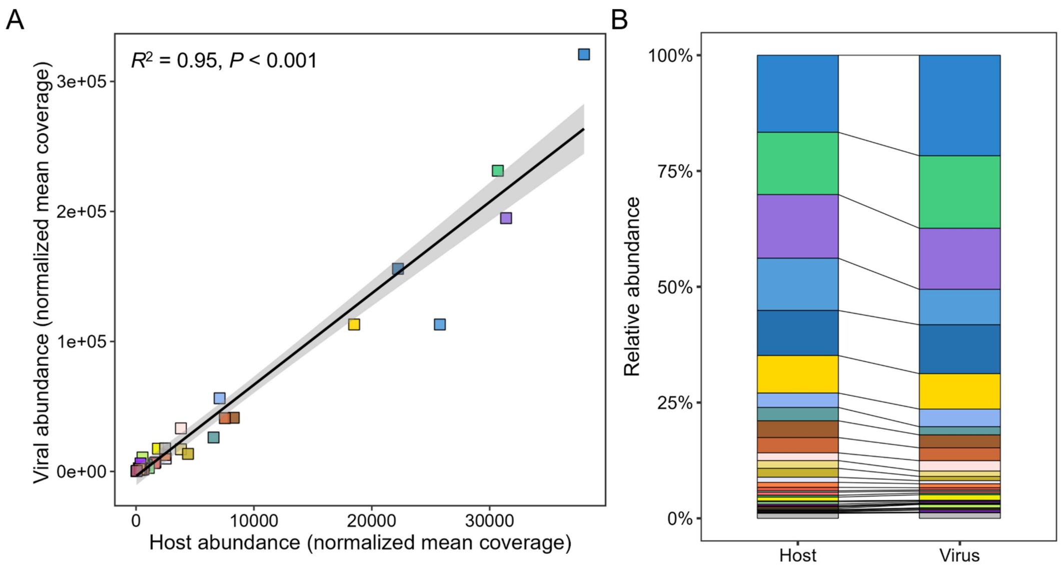


**Fig. S8 Host-linked viral abundance in AS. A** Correlation analysis between the abundance (normalized mean coverage) of viral operational taxonomic units (vOTUs) and their predicted prokaryotic hosts. The gray shaded area shows 95% confidence interval of the fit. Different color represents various host phyla. **B** Relative abundances (%) of vOTUs and their predicted prokaryotic hosts grouped by the host taxonomy in AS. Each host phylum is represented by a different color.

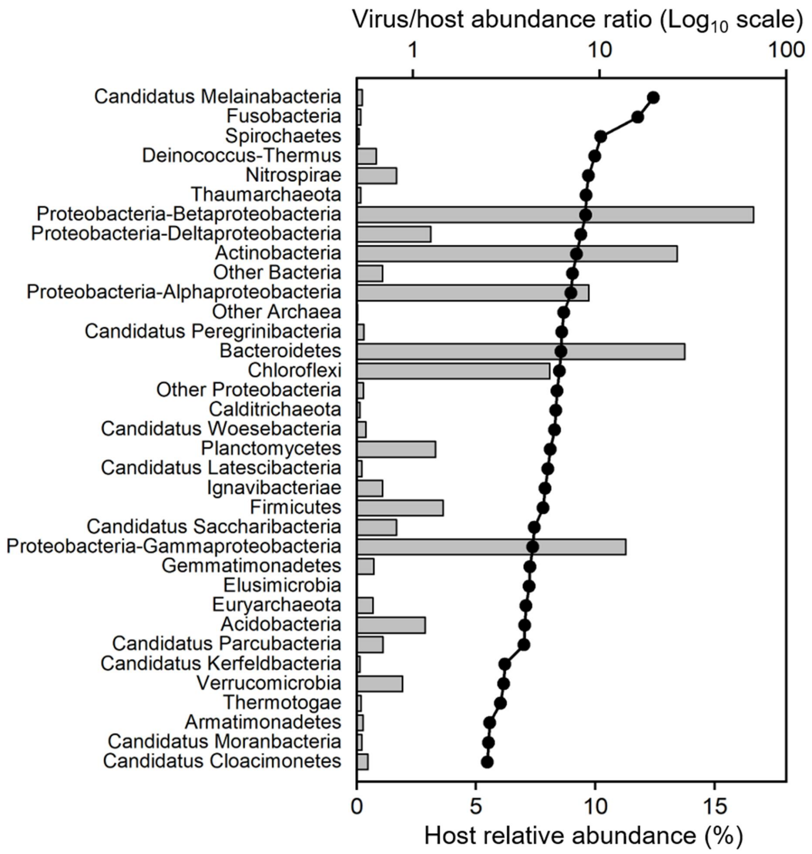


**Fig. S9 Virus/host abundance ratio and host relative abundance for all predicted hosts.**
